# Building Trust in Governmental and Educational Authorities in Adolescence: A Comparison of Early, Middle, and Late Adolescents in Four European Countries

**DOI:** 10.1007/s10964-025-02297-3

**Published:** 2025-12-04

**Authors:** Jakub Brojac, Jan Šerek, Simon Forstmeier, Ana Đorđević, Vujo Ilić, Enrico Padoan, Lenka Štěpánková, Anne Möbert, Anna Masling, Francesco Marangoni, Jana Fikrlova

**Affiliations:** 1 Masaryk University, The Psychology Research Institute, Faculty of Social Studies, Joštova 218/10, Brno, 60200 Czech Republic; 2 University of Siegen, Department of Psychology, Adolf-Reichwein-Str. 2a, Siegen, 57076 Germany; 3 University of Belgrade, Kraljice Natalije 45, Institute for Philosophy and Social Theory, Beograd, 11000 Serbia; 4 University of Siena, via Fieravecchia, 19, Department of Social, Political and Cogitive Sciences, 53100 Siena, Italy

**Keywords:** Trust, Procedural justice, Adolescence, Institutional trust, Cross-national comparison

## Abstract

Research on procedural justice shows that giving voice to citizens or experts, providing transparent explanations, and acting predictably fosters trust in authorities. Yet little is known about how these processes operate during adolescence. It remains unclear whether these aspects of authority behavior are relevant to trust-building at the onset of adolescence and how their importance evolves with age. This study reports findings from a cross-national vignette experiment involving 2383 adolescents aged 11–12 (31% of the sample), 14–15 (33%), and 18–19 (36%) from Germany (*n* = 608, 55% female), Italy (*n* = 426, 44% female), Czechia (*n* = 724, 51% female), and Serbia (*n* = 625, 60% female). Results showed that voice and transparency in rationale were the strongest predictors of trust in both school and governmental authorities. In most cases, these effects did not vary significantly with age. Even early adolescents were responsive to procedural justice aspects. Consistent age-related moderations appeared only in the post-communist countries of Czechia and Serbia, where the effects of voices and transparency of rationale on trust increased with age. This suggests that contextual factors can shape adolescents’ developing capacity to evaluate authority behavior. These findings show that adolescents already have the capacity to value procedural justice from early adolescence. However, the delayed effects in post-communist countries underscore the importance of the socio-political context, which may shape the opportunity to learn and apply these procedural justice judgments when forming trust in authorities.

**Supplementary Information:**

The online version contains supplementary material available at 10.1007/s10964-025-02297-3.

## Introduction

When adolescents trust authorities to act benevolently and in the common interest, it benefits both them and society at large. While uncritical trust can be as problematic as extreme distrust, a certain level of trust is necessary to achieve cooperation and positive outcomes for adolescents’ development. For instance, trust in teachers predicts adolescents’ ability to learn (Landrum et al., 2015), promotes greater civic engagement (Torney-Purta et al., 2004), and enhances compliance with laws and public health measures, as seen during the COVID-19 pandemic (Marien & Hooghe, 2011; Bulut & Samuel, 2023). To achieve these positive outcomes, it is essential to understand the specific authority behaviors that build or erode trust among adolescents. Previous research, largely inspired by the procedural justice approach, has identified several key aspects of an authority’s behavior as important for building trust: providing voice to individuals, maintaining transparency, or acting predictably (Blader & Tyler, 2003; Tyler, 2012). However, this research often focuses on adults and pays limited attention to the developmental perspective (Granot & Tyler, 2019; Tyler & Trinkner, 2017). It remains largely unknown whether these specific authority behaviors are equally relevant to trust-building at the onset of adolescence and how their importance might shift on the path to adulthood. This study addresses this gap by using a cross-country vignette experiment to examine how procedurally just behavior influences trust among early, middle, and late adolescents, offering an understanding of how authorities can build trust throughout this developmental period.

### The Procedural Justice Approach To Trust Building

Trust can be understood as one’s willingness to accept vulnerability to the acts of another party in an uncertain situation, stemming from the expectation that the other party will act in one’s interest (Mayer et al., 1995; Thielmann & Hilbig, 2015). In the context of authority, trust is a psychological state closely related to perceptions of legitimacy and the willingness to accept decisions, recommendations, or norms issued by the authority (Hamm et al., 2017). Many sources influence the trust in and perceived legitimacy of authorities’ decisions. These may include sociocultural aspects of trustors’ lives (Dinesen & Bekkers, 2017; Kaasa & Andriani, 2022), individual differences among trustors, such as generalized dispositional trust or betrayal sensitivity (Thielmann & Hilbig, 2015), the perceived fairness of resource distribution (Esaiasson et al., 2019; Walster & Walster, 1975), or the perceived procedural justice (Blader & Tyler, 2009; Tyler & Blader, 2003).

The key aspect of procedural justice is that it can be adjusted. This contrasts with outcome favorability, which authorities often cannot achieve perfectly (Gomberg, 2016). In fact, procedural factors are especially important in situations where generally acceptable outcomes cannot be achieved (Šerek et al., 2022). According to the procedural justice approach, people view authorities as legitimate and trust them based on observing their actual behavior and assessing their fairness (Tyler, 2012). Two fundamental intertwined components of procedural fairness can be differentiated: the quality of decision-making, referring to how decisions are made, and the quality of interpersonal treatment, referring to how people feel treated during the procedure (Blader & Tyler, 2003; Tyler & Blader, 2000). Practical manifestations of the quality of decision-making include whether people are given the opportunity to present their perspectives during the decision-making process or whether authorities decide impartially based on rules and facts. The quality of interpersonal treatment can be manifested by showing care for people’s situations, concerns, and needs, or showing respect for their rights and status within society (Tyler, 2012). Nevertheless, in practice, it can be difficult to distinguish the effects of these factors in the decision-making process. For example, when authorities give people a voice during decision-making and allow them to participate in the process, this can be perceived both as enhancing the perceived quality of the decision-making and as a sign of interpersonal respect from the authority (Blader & Tyler, 2003; Tyler, 2012).

Inspired by procedural justice theory, four specific aspects of authorities’ behavior are known to positively affect how people perceive authorities: voice of citizens, voice of experts, transparency in rationale, and a predictable framework. The concept of *voice* refers to the ability of people (or experts) to influence decision-making outcomes (Tyler, 2012). Beyond direct control over outcomes, individuals often care about the symbolic, non-instrumental aspects, particularly the opportunity to express their views, even when that expression is unlikely to influence the final decision (Platow et al., 2006, 2013). This applies to a realistic form of voice in school and governmental decision-making. Citizens or experts often do not have direct control over the outcome, but authorities might consider their views. Furthermore, authorities are often expected to exhibit at least some level of *transparency*, which can take two forms: transparency in process and transparency in rationale (de Fine Licht & Naurin, 2022; Mansbridge, 2009). While the former refers to transparency about the details of the decision-making process, the latter refers to transparency about the information, facts, and reasons on which the decisions are based. Because transparency in process is sometimes viewed as impractical and potentially having unintended consequences, transparency in rationale may be perceived as a preferable alternative. Lastly, providing a *predictable framework* means that authorities are clear about how their decisions will be implemented, what the timeline will be, and under what conditions the decisions can change. By doing this, authorities acknowledge people’s need for a predictable environment, show them respect, and allow them to plan their activities and prepare for the consequences of authorities’ actions (Tyler, 2012). For example, especially during the highly chaotic times of the COVID-19 pandemic, the predictability and stability of authorities’ actions was an important source of political trust (Weinberg, 2022).

### The Development of Trust in Authorities Over Adolescence

The ways in which the above-mentioned aspects of authorities’ decision-making translate into trust can differ from early to middle to late adolescence. The main presumed reasons are cognitive developmental changes occurring during this life period. While the positive effect of perceived procedural justice on trust in authorities remains invariant with age during adulthood (Wolfe et al., 2016), adolescence is characterized by the gradual improvement of social learning skills, perspective-taking abilities, and the ability to combine different perspectives, as well as the growing complexity and abstractness of thinking (Blakemore & Mills, 2014; Steinberg, 2005). Consequently, it is proposed that these cognitive changes can affect how trust in authorities is formed.

First, compared to childhood, there are changes in how adolescents perceive trust and develop trusting relationships. Specifically, higher perspective-taking abilities in adolescence are associated with higher levels of trust toward trustworthy counterparts and a stronger decline in trust toward untrustworthy counterparts (Fett et al., 2014). Consequently, one’s certainty about others’ behavior decreases during early adolescence, which leads to relying less on prior beliefs and more on sampled evidence. Mid-adolescents become more capable of assessing that some people are more trustworthy than others and considering a broader set of hypotheses about others’ trustworthiness. The increase in uncertainty among 13-15-year-olds (compared to 10-12-year-olds) is also associated with becoming more tolerant of uncertainty (Ma et al., 2022). Furthermore, early adolescents do not distinguish between interpersonal trust in friendships and generalized interpersonal trust as clearly as late adolescents, whose beliefs about the world are more crystallized (Wray-Lake & Flanagan, 2012). Taken together, trust becomes increasingly nuanced and evidence-based during adolescence, which might lead to greater attention paid to specific aspects of authorities’ behavior and a greater impact of them on trust.

Additionally, as adolescents’ reasoning becomes more complex, they grow increasingly critical of authority figures. Adolescence is a crucial period for identity formation, marked by questioning parental decisions, reducing parental control, and developing individual decision-making skills (Benson & Elder Jr., 2011; Cumsille et al., 2006; Koepke & Denissen, 2012). As they mature, adolescents gain more opportunities to form autonomous opinions and directly experience various aspects of decision-making by interacting with diverse authorities in different environments (Koepke & Denissen, 2012). Thus, adolescents gradually become more cautious about adults and discover they can’t take anyone at face value (Noam et al., 2013). The developing autonomy and broader experiences enable older adolescents to question the authority of teachers in schools, especially if teachers do not provide support for autonomy to those who perceive themselves as more autonomous (Graça et al., 2013).

Furthermore, older adolescents tend to trust institutions less than their younger counterparts (Torney-Purta & Amadeo, 2003) and are more inclined to scrutinize the credibility and expertise of sources before accepting information (Mann et al., 1989). As they age, adolescents become better able to evaluate their perspectives within a broader social context, allowing them to consider complex aspects of the social world (Blakemore & Mills, 2014). This enables them to critically evaluate the trustworthiness of political and legal authorities based on the (in)congruence of these authorities’ behaviors with their own gradually developed political attitudes (Tyler & Trinkner, 2017). Overall, it seems plausible that older adolescents evaluate individual authorities more critically through the lenses of more complex cognitive reasoning, acquired social, moral norms, and developed identity.

Despite the evidence indicating that older adolescents can be more sensitive to how authorities proceed when making their decisions than younger adolescents, previous research also provides some support for the opposite hypothesis. That is, early adolescents already sufficiently consider factors such as voice or transparency and translate them into their trust. For example, research rooted within social domain theory has provided robust evidence that three-to-four-year-olds are already able to understand that authorities’ commands can contradict moral norms (Smetana & Yoo, 2022; Yoo & Smetana, 2022). In addition, the tendency to judge behaviors of authorities as unfair and resist them becomes more pronounced from the age of four to 10 (Smetana et al., 2021). Thus, although social domain research does not explicitly focus on procedural aspects of decision-making, it seems that early adolescents can identify and critically evaluate authorities’ problematic behaviors. This is consistent with the procedural justice approach, according to which perceptions of procedural justice might predict the perceived legitimacy of authorities not only among adults but also among adolescents (Fagan & Tyler, 2005; Tyler & Trinkner, 2017). However, the direct empirical evidence supporting this is scarce, lacking information on the overall effect of procedural justice in adolescence as well as on the differences between its various stages.

### The Role of Context in Adolescents’ Trust in Authorities

Adolescents’ trust varies across different authorities, and the processes through which they develop this trust may also differ. Adolescents show greater confidence in social authorities (e.g., schools, religious institutions) than in legal ones (e.g., law enforcement, the justice system; Fine et al., 2019; Nivette et al., 2022; Sears & Brown, 2023; Tyler & Trinkner, 2017). Moreover, the actions of one authority can influence perceptions of another. For instance, fair treatment by teachers can lead to viewing police as more legitimate (Nivette et al., 2022). Trust in accessible authorities, such as parents and teachers, may shape adolescents’ perceptions of more distant authorities like police officers and politicians (Cavanagh & Cauffman, 2019; Nivette et al., 2022; Sears & Brown, 2023). This suggests that trust in different types of authorities develops at different rates. Initially, adolescents develop trust in proximate authorities, and over time, trust in more distant authorities develops as well, influenced in part by broader societal interactions and experiences (Sears & Brown, 2023; Tyler & Trinkner, 2017). Consequently, the procedural behavior of authorities can affect adolescents’ trust in varying ways, depending on the authority involved. For instance, political or governmental decision-making contexts may be harder for younger adolescents to evaluate, potentially reducing the impact of procedurally just or unjust behavior by political authorities on their trust.

Additionally, the effects of procedural factors on trust may differ across sociocultural contexts. In countries with lower overall trust levels, adolescents may become sensitive to negative actions by authorities at an earlier age. This “sensitization” hypothesis suggests that in low-trust contexts, where negative behaviors such as corruption and lack of transparency are more prevalent, even younger adolescents are more likely to encounter and recognize such practices. However, the opposite “desensitization” effect is also possible. In settings where fair behavior by authorities is less common, adolescents may adopt a prevailing norm of distrust and, due to limited exposure to fair treatment, become less able to recognize and evaluate fairness (Nivette et al., 2022).

In a similar vein, adolescents’ responsiveness to procedural aspects of authority behavior may be shaped by broader patterns of political socialization within a country. Specifically, the extent to which socialization agents such as families and schools encourage young adolescents to openly discuss sociopolitical issues and develop independent, critical perspectives on authorities plays a crucial role in political socialization (Campbell, 2008; Schmid, 2012). As a result, in contexts where younger adolescents receive little support from socialization agents to engage with or reflect on sociopolitical issues, their ability to translate procedural aspects of authority behavior into trust may emerge only later in development. Such “delayed” political socialization may occur, for example, in post-communist countries, where hierarchical status roles and deference to authority remain more deeply entrenched, and youth participation in public affairs is less valued by society (Klicperová et al., 1997).

## The Current Study

Research has consistently shown that procedural justice fosters trust in authorities. However, it remains unclear whether procedural justice aspects are relevant for building trust already at the beginning of adolescence and how their importance might evolve with age. Addressing this gap, this study investigates how procedurally just behavior impacts trust among early, middle, and late adolescents. In the current study, it was expected that giving voice to citizens, giving voice to experts, being transparent in rationale, and providing a predictable framework would increase adolescents’ trust in authorities (H1a-d). Moreover, it was examined whether the presumed effects differed among early (ages 11–12), middle (ages 14–15), and late (ages 18–19) adolescents. Based on the literature, two alternative hypotheses were considered: either older adolescents are more sensitive to the presence of the four procedural aspects of authority behavior than younger adolescents (H2, a positive moderation by age), or young adolescents already acknowledge these procedural aspects, meaning the expected effects would appear in all stages of adolescence (i.e., no moderation by age). To ensure the robustness of the findings, the presumed effects were tested for two types of authority (school-level and governmental) using four independent samples from different European countries: Czechia, Germany, Italy, and Serbia. These countries differed in important sociocultural characteristics, such as average levels of institutional trust: Germany represented a relatively high-trust context, Italy and Czechia moderate-trust contexts, and Serbia a low-trust context (European Social Survey, 2023). Moreover, Germany and Italy represented Western democracies with long-established democratic traditions, whereas Czechia and Serbia represented post-communist contexts. Although the existing literature does not allow for convincing predictions regarding cross-country differences, it was expected that moderation by age (H2), if present, could be more pronounced in the governmental context and in post-communist countries.

## Method

### Participants and Sample Size

Participants were recruited through panels provided by professional companies (all participants in Germany and Italy, and participants aged 11–12 and 14–15 in Serbia), public schools (participants aged 11–12, 14–15, and some of those aged 18–19 in Czechia), or snowballing and advertising on social media networking sites (participants aged 18–19 in Serbia and some in this age group in Czechia). The research has been reviewed and approved by the research ethics committees of the participating universities. Data collection among minors was contingent on the consent of their parents. Only anonymous quantitative data were collected. The data collection took place in spring 2023.

The aim was to recruit 200 respondents per country for each age group, resulting in a total of 600 respondents per country. This sample size was expected to provide sufficient power (0.80) to detect age moderation, assuming no effect of experimental conditions among early adolescents, a small effect (0.2 SD) among middle adolescents, and a large effect (0.8 SD) among late adolescents (calculated using the R Superpower package, Lakens & Caldwell, 2021; see Supplementary Material for details). To avoid potential bias due to participants who paid only limited attention to the experimental materials (Huang et al., 2015), those who completed the questionnaire in less than four minutes were excluded. This was determined to be the minimum necessary time for reading the vignettes and answering the items based on the cognitive interviews and careless response pattern analysis (Gottfried et al., 2022).

The final cross-national sample included 2,383 adolescents. The target of recruiting at least 200 participants per age group was achieved in all countries except Italy. Sample sizes were 608 in Germany (202/201/205), 426 in Italy (120/160/146), 724 in Czechia (215/231/278), and 625 in Serbia (210/200/215), with the numbers in parentheses indicating the subgroups aged 11–12, 14–15, and 18–19, respectively. The proportion of women was 55% in Germany, 44% in Italy, 51% in Czechia, and 60% in Serbia. Adolescents from ethnic minority backgrounds accounted for 9% of the sample in Germany, 3% in Italy, 5% in Czechia, and 4% in Serbia.

### Procedure

All participants completed online questionnaires, except for those recruited through schools, who completed printed versions. The questionnaire included items on generalized interpersonal trust and an experimental component.

In the experimental section, every participant was presented with two vignettes. The first vignette regarded the school context and described a hypothetical situation in which school management was deciding on the ban of cell phones among students. The second vignette was related to the national context and described a hypothetical situation in which the government was deciding on introducing anti-pandemic measures. Immediately after reading each vignette, participants completed a scale asking how much they would have trusted the described authority (i.e., the school management or the government).

Each vignette had 12 variants that systematically varied in whether (1) voice was given to citizens, experts, or nobody (i.e., three levels), (2) transparency in rationale was present or absent (i.e., two levels), and (3) a predictable framework was provided or not (i.e., two levels). One variant of the school vignette and one variant of the governmental vignette were randomly assigned to every participant (the randomization of the vignettes was independent from each other). The full wording of the vignettes in English can be found in Table 1 (translations into national languages are available in the Supplementary Material).

**Table 1:** Full wording of the vignettes in english

School vignette			
Introduction			
*Imagine that you visit a school where several cases of cyberbullying using mobile phones have happened recently. Some students used their phones to mock and humiliate others. Students*,* teachers*,* and parents think that the school management has to do something because the bullying has been very serious and might happen again and again. Therefore*,* the school management decides to take tough steps. They decide to impose a ban on using mobile phones. Students are not allowed to use their mobile phones in classrooms under a penalty. This decision is announced to students at a joint meeting with the management.*			
Voice			
Citizens	Experts		None
*The management are interested in students’ opinions before taking a decision. When deciding*,* they collect students’ opinions using anonymous online polls or pieces of paper and consider students’ opinions carefully.*	*The management are interested in expert opinions before they take a decision. When deciding*,* they approach academic experts in cyberbullying*,* youth workers*,* and exeprienced teachers. The ban on using phones is thoroughly consulted with these experts.*		*The management decide on the measures by themselves. They are not interested in the opinions of citizens or experts and do not take them into account when making decisions.*
Transparency in rationale			
Yes		No	
*The management do their best to explain the decision carefully. The management shed light on all reasons*,* arguments*,* and facts that have been considered. They make clear why they prefer the current solution over other possibilities.*		*The management do not explain their decision in any way. They present to the students only their final decision but not the specific reasons based on which the decision has been made. They also do not explain why such a solution has been chosen over other options.*	
Predictable framework			
Yes		No	
*The management wants to give students some certainty. They tell students a clear plan on when the ban will be imposed*,* how the ban will be monitored*,* and what the penalty will look like. This helps students to prepare in advance.*		*The management present their decision saying that it will probably change continuously. Students thus do not know a clear plan of what measures will be taken and when. It is also difficult for students to prepare for the measures in advance.*	

Government vignette			
Introduction			
*Imagine that another pandemic of infectious disease is coming to our country. It can be a strong flu or another variant of covid. The government have to take action. It seems that the mandatory testing and wearing of facemasks in some places are the most effective moves at this moment. Therefore*,* the government assemble and decide on specific places where facemasks will be mandatory. They also determine in which situations people have to take tests. All measures are first announced in the evening television news by the health minister.*			
Voice			
Citizens	Experts		None
*The government are interested in citizens’ opinions before taking a decision. When deciding*,* they carefully consider all public opinion polls on this issue and petitions from ordinary citizens.*	*The government are interested in expert opinions before they take a decision. When deciding*,* they assemble a board of experts in epidemiology*,* virology*,* sociology*,* economy and related disciplines. All measurers are thoroughly consulted with these experts.*		*The government decide on the measures by themselves. They are not interested in the opinions of citizens or experts and do not take them into account when making decisions.*
Transparency in rationale			
Yes		No	
*The government do their best to explain the decision carefully. The government shed light on all reasons*,* arguments*,* and facts that have been considered. They make clear why they prefer the current solution over other possibilities.*		*The government do not explain their decision in any way. They present to the citizens only their final decision but not the specific reasons based on which the decision has been made. They also does not explain why such a solution has been chosen over other options.*	
Predictable framework			
Yes		No	
*Even though the situation can suddenly change*,* the government want to give people some certainty. They present people with a clear plan*,* showing them what measures will be taken and when. This helps people to prepare in advance.*		*The government present their decision saying that it will probably change continuously. People thus do not know a clear plan of what measures will be taken and when. It is also difficult for people to prepare for the measures in advance.*	

Every vignette was composed of the introduction, one level of the voice factor, one level of the transparency factor, and one level of the predictable framework factor (always in this ordering)

### Measures

All measures had five-point response scales from “strongly disagree” (= 1) to “strongly agree” (= 5) and the option “I can’t answer” (coded as missing). Total scores were created by averaging the items. Translations of all items into national languages are provided in the Supplementary Material.

### Trust in School Management and Trust in Government (outcome variables)

Trust was measured with four items partly adapted from Hamm et al. (2017, 2019). Depending on the vignette, the items referred either to school management or to the government: “I would be comfortable with being vulnerable to the judgement of such a government/management,” “I would be open to letting such a government/management make more decisions about issues that are important to me,” “I would be comfortable with letting such a government/management make more decisions that may affect my future,” and “I would expect that letting such a government/management make decisions won’t harm me.”). Internal consistency was good for both trust in school management (ω_GER_ = 0.87, ω_ITA_ = 0.83, ω_CZE_ = 0.83, ω_SRB_ = 0.88) and trust in government (ω_GER_ = 0.90, ω_ITA_ = 0.86, ω_CZE_ = 0.88, ω_SRB_ = 0.93). The reported internal consistency of trust in school management in Serbia was calculated using a three-item scale, as item 3 was excluded following measurement invariance testing (see below).

### Generalized Interpersonal Trust (control variable)

Trust in other people was measured using items taken from previous studies (Baltatescu, 2009; Couch et al., 1996; Yamagishi & Yamagishi, 1994). The final five items were selected based on a study by Zhang (2021): “Most people are trustworthy,” “Most people are basically good and kind,” “Most people are basically honest,” “Most people can be trusted,” and “Most of the time, people are helpful.” The scale had good internal consistency in all countries (ω_GER_ = 0.88, ω_ITA_ = 0.91, ω_CZE_ = 0.74, ω_SRB_ = 0.90).

### Analytic Strategy

All analyses were conducted separately for every country. The underlying data are accessible via OSF: https://osf.io/x9ajy/overview? view_only=13bb184e439543c4b775fc9e41d42a7c. The scripts used to perform the analyses are available in the Supplementary Material.

Before conducting the main analysis, the measurement invariance of outcome variables across the three age groups was examined. A two-factor model was specified representing trust in school management and trust in government, with each factor indicated by four items. Correlations were allowed between the corresponding items of the two factors. To assess invariance, models assuming configural, metric, and scalar invariance were compared, using the recommended cutoff values (ΔCFI and ΔTLI ≤ 0.01; ΔRMSEA ≤ 0.015; Cheung & Rensvold, 2002; Putnick & Bornstein, 2016). When evidence of non-invariance was detected, modification indices were inspected to identify the specific constraints responsible. All analyses were conducted in Mplus 8.11 (Muthén & Muthén, 2017) using the WLSMV estimator, which is appropriate for Likert-type indicators.

To account for individual differences, generalized interpersonal trust and gender were included as control variables. Generalized interpersonal trust reflects a baseline that individuals use particularly when evaluating novel situations (Rotter, 1971) and has been shown to decline over adolescence (Wray-Lake & Flanagan, 2012). This variable was therefore controlled for to better isolate the effects of the experimental vignettes. In addition, because gender differences in trust and trusting behavior have been observed during adolescence (Derks et al., 2014; Wray-Lake & Flanagan, 2012), adolescents’ gender was also controlled for to account for potential gender imbalances across subsamples.

The hypotheses were tested using factorial ANCOVA. Separate models were estimated for the school vignette and the governmental vignette. Each model included five categorical predictors (factors): three independent variables representing the experimental manipulations – voice (three levels), transparency of rationale (two levels), and predictable framework (two levels); a moderator variable – age group (three levels); and a control variable – gender (four levels: female, male, other, unspecified). In addition, generalized interpersonal trust was included as a continuous covariate. In the first step, a model without interactions was tested to assess main effects of experimental conditions (Model 1). The model was then extended by including three two-way interactions between age group and the three independent variables to assess whether their effects were moderated by age (Model 2). Contrasts between experimental conditions across age groups were plotted. The analysis was performed in R (version 4.4.3; R Core Team, 2024). Due to occasional missing values, 97–99% of participants were included in the models depending on the country.

## Results

### Measurement Invariance across Age Groups

Full metric and scalar invariance were established in Germany, Italy, and Czechia, as changes in CFI, TLI, and RMSEA remained well below the recommended cutoff values. In contrast, Serbia did not meet the criteria for metric or scalar invariance (ΔRMSEA > 0.015). Further analysis identified one item from the scale of trust in school management (item 3) as the primary source of this discrepancy. After removing this item, metric invariance was achieved. However, only partial scalar invariance was observed: the intercepts of two items assessing trust in government (items 1 and 2) and one item assessing trust in school management (item 4) had to be freed in the oldest age group (Table 2).

Based on these results, the overall score of trust in school management for the Serbian sample was calculated using only three items. Additionally, comparisons of mean trust levels between the oldest and the two younger age groups in Serbia should be interpreted with caution. Nonetheless, the establishment of metric invariance allowed for valid cross-age comparisons of the effects of experimental conditions on trust, which was the primary objective of this study.

**Table 2:** Measurement invariance testing

	χ^2^[df]	CFI	TLI	RMSEA	Δχ^2^[df]
Germany					
Configural	53.011[45]	0.997	0.994	0.030	
Metric	61.851 [57]	0.998	0.997	0.021	8.840[12]
Scalar	83.969[69]	0.994	0.993	0.033	22.118[12]*
Italy					
Configural	65.063[45]*	0.988	0.978	0.056	
Metric	80.581[57]*	0.986	0.979	0.054	15.518[12]
Scalar	95.598[69]*	0.984	0.981	0.052	15.017[12]
Czechia					
Configural	80.718[45]**	0.986	0.974	0.057	
Metric	96.963[57]**	0.985	0.977	0.054	16.245[12]
Scalar	118.534[69]**	0.981	0.977	0.055	21.572[12]*
Serbia					
Configural	47.027[45]	0.999	0.999	0.015	
Metric	80.366[57]	0.993	0.990	0.044	33.340[12]**
Scalar	175.330[69]	0.963	0.970	0.086	94.963[12]**
Serbia (revised)					
Configural	35.068[30]	0.998	0.996	0.029	
Metric	51.136[40]	0.996	0.994	0.037	16.068[10]
Scalar	126.889[50]**	0.973	0.966	0.086	75.752[10]**
Partial scalar	69.092[47]*	0.992	0.990	0.048	17.955[7]*

Item 3 from the scale measuring trust in school management was omitted in the revised model in Serbia. The partial scalar model has unconstrained intercepts for items 1 and 2 measuring trust in government and item 4 measuring trust in school management

** *p*  < .01. * *p*  < .05

### Descriptive Analyses

Mean levels of the outcome variables across age groups and experimental conditions are shown in Table 3. The descriptive results indicated that reported trust was most often lower among older adolescents. Differences between experimental conditions aligned with expectations: trust levels were higher when participants were exposed to conditions involving voice, transparency in rationale, or a predictable framework. The two outcome variables were positively correlated in all countries except Czechia (r_GER_ = 0.24, r_ITA_ = 0.47, r_CZE_ = 0.02, r_SRB_ = 0.45).

**Table 3:** Trust in school management and government by age groups and experimental conditions (Means and standard Deviations)

	Trust in school management												Trust in government										
	Germany			Italy			Czechia			Serbia			Germany			Italy			Czechia			Serbia	
	M (SD)	n		M (SD)	n		M (SD)	n		M (SD)	n		M (SD)	n		M (SD)	n		M (SD)	n		M (SD)	n
Age group																							
11–12	3.30 (0.94)	198		3.53 (1.05)	119		2.77 (0.72)	212		3.38 (0.99)	206		2.97 (0.98)	191		3.39 (1.18)	118		2.82 (0.79)	213		3.36 (0.92)	207
14–15	3.27 (0.96)	198		3.18 (1.01)	158		2.62 (0.80)	231		3.06 (1.08)	200		3.22 (1.06)	199		3.26 (1.03)	157		2.83 (0.86)	228		2.97 (1.06)	198
18–19	2.79 (0.78)	204		2.76 (0.80)	144		2.40 (0.91)	277		2.42 (0.97)	212		2.95 (0.92)	205		3.09 (0.86)	145		2.77 (1.02)	277		2.43 (1.09)	205
Voice																							
Citizens	3.20 (0.82)	195		3.25 (0.95)	141		2.74 (0.84)	236		3.05 (1.09)	197		3.21 (0.97)	196		3.42 (1.02)	152		3.01 (0.91)	230		3.14 (1.05)	214
Experts	3.24 (0.94)	203		3.23 (1.03)	131		2.62 (0.81)	251		3.11 (1.03)	202		3.17 (0.93)	208		3.17 (0.97)	144		2.95 (0.87)	234		2.93 (1.05)	201
None	2.91 (0.98)	202		2.92 (0.98)	147		2.36 (0.82)	231		2.69 (1.10)	219		2.74 (1.02)	191		3.09 (1.07)	124		2.48 (0.84)	254		2.66 (1.13)	195
Transparency in rationale																							
Yes	3.28 (0.90)	301		3.21 (1.02)	222		2.74 (0.82)	355		3.10 (1.02)	301		3.19 (0.95)	308		3.37 (0.98)	218		3.03 (0.88)	369		3.04 (1.03)	313
No	2.96 (0.93)	299		3.03 (0.96)	197		2.42 (0.82)	363		2.79 (1.14)	317		2.89 (1.02)	287		3.09 (1.05)	202		2.56 (0.87)	349		2.79 (1.14)	297
Predictable framework																							
Yes	3.16 (0.91)	305		3.25 (1.02)	216		2.66 (0.80)	354		3.11 (1.07)	308		3.18 (0.96)	286		3.30 (1.00)	195		2.91 (0.89)	366		2.99 (1.10)	304
No	3.07 (0.95)	295		3.00 (0.96)	203		2.50 (0.86)	364		2.78 (1.08)	310		2.92 (1.01)	309		3.19 (1.05)	225		2.69 (0.91)	352		2.85 (1.08)	306

Generalized interpersonal trust, which served as a control variable, was highest in Germany, M(SD)_GER_ = 3.15(0.76), M(SD)_ITA_ = 2.94(0.94), M(SD)_CZE_ = 2.76(0.61), M(SD)_SRB_ = 2.92(0.95). It was positively correlated with both trust in school management (r_GER_ = 0.19, r_ITA_ = 0.43, r_CZE_ = 0.15, r_SRB_ = 0.36) and trust in government (r_GER_ = 0.26, r_ITA_ = 0.39, r_CZE_ = 0.09, r_SRB_ = 0.32), although the strength of these associations varied across countries.

### The Effects of Experimental Conditions on Trust

Testing models without interactions generally confirmed the expected positive effects of voice, transparency in rationale, and a predictable framework on trust (H1a–d; Table 4). Voice effects emerged for both types of authority across all countries, ranging from small to medium in size. Post-hoc tests with Bonferroni correction indicated that both citizen voice and expert voice conditions led to significantly higher trust compared to the no voice condition (ps < 0.05), while the difference between citizen and expert voice was not significant. This pattern held for both types of authority in all countries except Italy, where the expert voice condition did not differ significantly from the no voice condition. Transparency in rationale also showed consistent small-to-medium effects across contexts. By contrast, the effects of a predictable framework were weaker, with small and occasionally nonsignificant effects (in Germany for the school context and in Italy for the governmental context).

**Table 4:** Factorial ANCOVA models predicting trust in school management and government (Partial η^2^)

	Trust in school management					Trust in government			
	Germany	Italy	Czechia	Serbia		Germany	Italy	Czechia	Serbia
*Model 1 (without interactions)*									
Voice	0.03**	0.02**	0.04**	0.04**		0.04**	0.03**	0.08**	0.03**
Transparency in rationale	0.03**	0.02**	0.04**	0.02**		0.03**	0.02**	0.08**	0.01**
Predictable framework	0.01	0.01*	0.01**	0.03**		0.02**	0.00	0.02**	0.01*
Age group	0.04**	0.02*	0.03**	0.06**		0.01*	0.01	0.00	0.06**
Gender	0.02**	0.12**	0.02**	0.05**		0.00	0.01	0.00	0.01
Generalized interpersonal trust	0.02*	0.00	0.00	0.02**		0.06**	0.14**	0.02**	0.03**
*Model 2 (with interactions)*									
Voice * Age group	0.01	0.01	0.01	0.00		0.00	0.03*	0.03**	0.02**
Transparency in rationale * Age group	0.00	0.02*	0.01*	0.01*		0.01	0.00	0.01*	0.04**
Predictable framework * Age group	0.01	0.00	0.00	0.01		0.00	0.00	0.01	0.00

Only interaction effects are shown for Model 2. Full ANCOVA results are provided in the Supplementary Material

** *p*  < .01. * *p*  < .05

### The Moderation of the Effects by Age

After introducing interactions into the models, significant age moderation was observed for transparency in the school context (three countries), transparency in the governmental context (two countries), and voice in the governmental context (three countries; Table 4). Further analyses, which disentangled the effects of citizen and expert voice by comparing each to the no voice condition (i.e., F-tests testing differences of predefined contrasts across age groups), revealed significant age interactions with citizen voice in Serbia and with expert voice in Czechia (ps < 0.05). Other interactions were not significant, indicating that in Italy, although there was a significant overall interaction between age and voice, neither citizen nor expert voice was convincingly moderated by age.

**Fig. 1:**
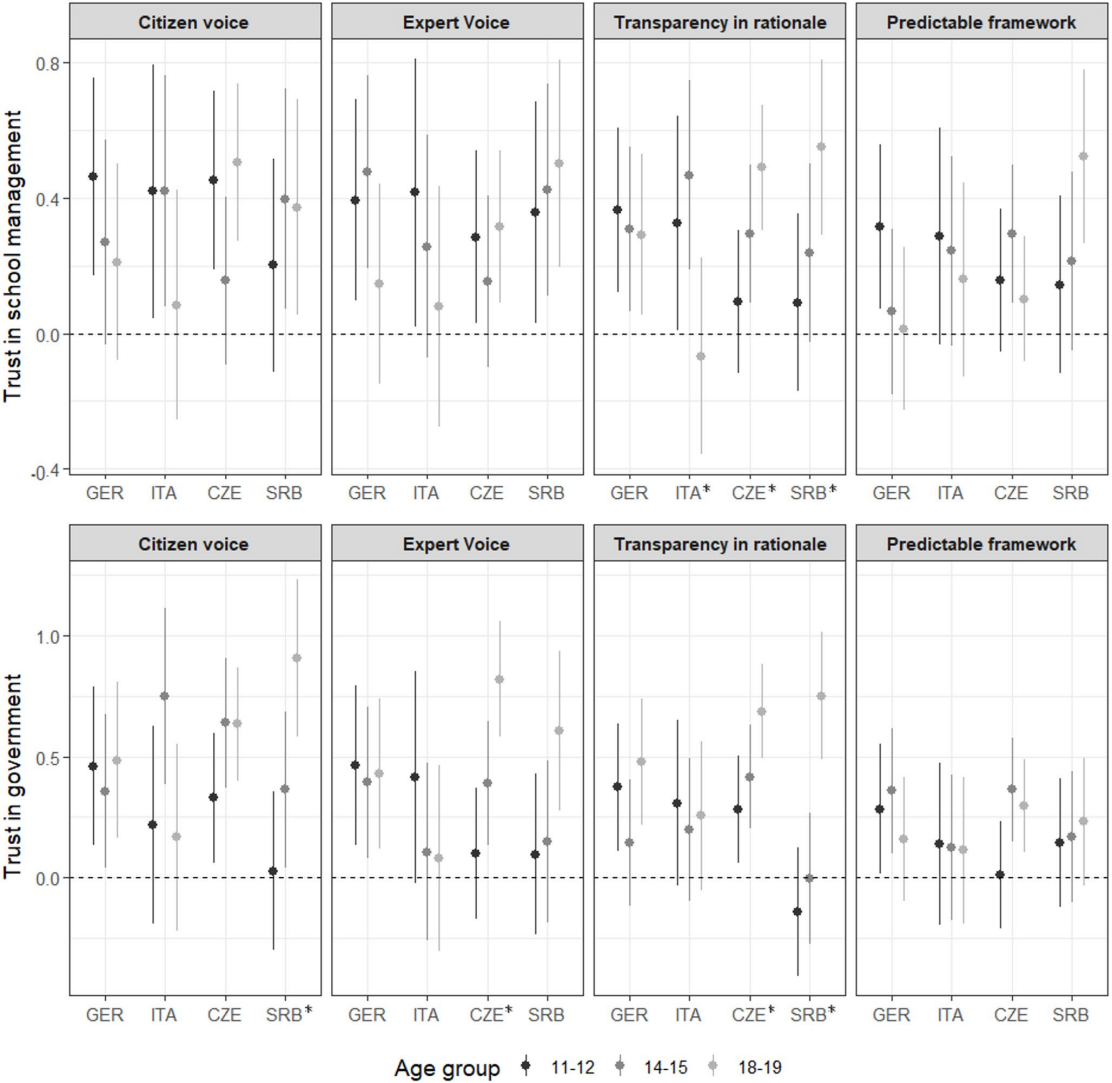
The Effects of Experimental Conditions by Age with 95% Confidence Intervals. Significant cross-age differences are marked with an asterisk. Parameter estimates and confidence intervals underlying this plot are provided in the Supplementary Material

Figure 1 presents a detailed investigation of age differences. The plots depict the effects of experimental conditions across age groups. While patterns varied across countries and different aspects of authority behavior, several general trends emerged. Even among the youngest adolescents, all procedural aspects of authority behavior were able to influence trust in at least some countries. This was particularly evident in Germany, where all examined aspects of authority behavior positively affected early adolescents’ trust in both school and governmental authorities. Next, among the seven significant interactions, six showed increasing effects with age and were observed in post-communist countries. Notably, four of these effects related to transparency in rationale, and four were observed in the governmental context. One significant interaction occurred in an unexpected direction: in Italy, the effect of transparency in rationale on school trust decreased with age.

## Discussion

Previous research has consistently shown that when authorities behave in accordance with the principles of procedural justice, it positively influences individuals’ trust in those authorities (Tyler, 2012). However, less attention has been paid to the developmental dimension of these effects. In particular, little is known about the extent to which early adolescents already take procedural justice into account, and whether its importance for trust formation increases from early to late adolescence. The present study addressed this gap by examining whether various aspects of procedural justice, such as giving voice to people and experts, ensuring transparency, and maintaining predictability, affect trust in school and governmental authorities among early, middle, and late adolescents. Findings from the cross-national vignette experiment indicated that even early adolescents are capable of considering procedural aspects of authority behavior when forming trust judgments.

The results showed that the procedural factors positively impacted adolescents’ trust in authorities. All four procedural factors increased trust in both school and governmental contexts, each procedural factor having its unique positive effects on higher trust. Transparency in rationale and the consideration of people’s or experts’ voices had consistent small to moderate effects on trust, while predictability had a comparatively lower but still statistically significant impact in most contexts. Additionally, the results demonstrated that the same procedural factors matter in both the proximal, everyday context of school and the more distant, abstract context of national government. These findings highlight the importance of procedural justice in shaping adolescents’ trust, particularly the roles of transparency and non-instrumental voices. Procedural aspects of authorities’ decision-making, which have been shown to foster adults’ trust, seem to be relevant also for adolescents (Blader & Tyler, 2003; Tyler & Trinkner, 2017). This is in line with research highlighting the sometimes-overlooked role of decision-making quality in shaping perceptions of legitimacy and institutional trust (Schmidt & Wood, 2019).

Only limited systematic age differences in the effects of procedural aspects on trust were found. In most contexts, the studied effects did not significantly vary with age. Even early adolescents aged 11–12 were often responsive to the presence or absence of the four aspects of procedural justice, a pattern particularly evident in the German data. Therefore, it seems that even younger adolescents are capable of evaluating the procedural dimension of authorities’ behavior. This is in line with the findings that children as young as three or four recognize that authority figures’ commands conflict with moral norms (Smetana & Yoo, 2022; Yoo & Smetana, 2022) and that the tendency to oppose such authority behaviors grows during childhood (Smetana et al., 2021). The present results expand this work by showing that early adolescents, at ages 11 or 12, attend to even more subtle aspects of authority behavior, such as transparency or predictability, and incorporate them into their trust judgments. It must be acknowledged that during adolescence, a critical view of authorities and institutions further develops, and trust becomes more sophisticated and evidence-based, particularly due to growing cognitive abilities and autonomy (Cumsille et al., 2006; Wray-Lake & Flanagan, 2012). Nevertheless, the capacity to form trust in authorities on the basis of perceived procedural fairness appears not to be contingent on these later developmental changes.

Yet, a limited set of age-related effects emerged. Most of them were in the expected direction, suggesting an increasing effect of procedural aspects on trust with age (the only exception was one negative age moderation found in Italy, which should be interpreted only cautiously given its sporadic nature). A closer inspection of the moderation pattern reveals that the positive moderation effects of age appeared only in post-communist countries with moderate or low institutional trust. They were observed most often, although not exclusively, for transparency in rationale and in the governmental context. Hence, despite the above-mentioned general capacity of younger adolescents to consider procedural aspects of authority behavior, it seems that its development might be shaped by contextual factors.

Specifically, it is possible that, in line with the idea of “desensitization”, adolescents may be less capable of recognizing fairness in contexts where authorities are generally perceived as unfair (Nivette et al., 2022). This might be particularly the case in Serbia, which is a low-trust country (European Social Survey, 2023) and widely classified as a hybrid or competitive authoritarian regime (Bieber, 2018; Levitsky & Way, 2020). In such contexts, adolescents’ political socialization may be delayed because family, school, and society tend to shield younger adolescents from engaging with negatively perceived social and political issues, thereby limiting their opportunities to learn procedural principles and apply them to broader political contexts (Nivette et al., 2022; Resh & Sabbagh, 2014; Schmid, 2012). Additionally, this tendency may be reinforced by a post-communist political culture marked by limited public participation and deference to authority. As a result, adolescents in these settings are likely to have fewer direct experiences with civic participation, collective decision-making, or public deliberation, both in schools and in the wider society. Thus, although they may already possess the cognitive capacity to consider procedural aspects of authority behavior, the scarcity of opportunities to develop and practice these skills may hinder the extent to which they integrate procedural fairness into their trust judgments.

It should be noted, however, that the moderation effects of age in Czechia and Serbia were relatively small, applied only to some procedural factors, and do not yield conclusive findings. Further research and replication are needed to clarify these context-specific patterns. In particular, it would be valuable to examine the extent of cross-national differences and to identify key factors that account for them, including elements of the supportive or protective societal climate and political culture that foster adolescents’ political socialization. Future research could also extend to pre-adolescent children in different countries, as it is possible that procedural factors lose their relevance across contexts at earlier developmental stages. Finally, the mechanisms by which children and adolescents transfer their evaluations of procedurally just authority behavior from one context to another warrant closer investigation.

This study has several limitations. First, due to its experimental design, the study was only able to examine the short-term effects of procedural factors. For instance, predictability appeared to play a less prominent role than other factors. However, this may be because the study’s design could not fully capture the impact of predictability, which likely unfolds over time. A longitudinal approach would be better suited to reveal how certain procedural elements influence long-term trust, which develops through adolescents’ repeated observations of authority behavior.

Second, the findings are based on adolescents’ evaluations of hypothetical scenarios. This assumes that participants can adequately imagine the situations and reflect on their expected perceptions. To strengthen ecological validity, future research should replicate these findings using naturalistic or quasi-experimental designs that capture real-world trust formation processes.

Third, although the study included adolescents from four European countries, the results may not generalize to regions with different political or social contexts, to youth from lower socioeconomic backgrounds, or to those with higher levels of political alienation, populist attitudes, or conspiratorial thinking. Current research suggests that such attitudes are becoming more common among adolescents and may significantly shape how this group perceives authority (Körner et al., 2023). Additionally, the Italian sample may have lacked sufficient statistical power, as the required sample size was not met.

Finally, the relatively subtle nature of the experimental manipulations may explain the small effect sizes. Stronger effects might emerge from more visible or morally severe procedural violations, or from repeated breaches. Nonetheless, the present findings indicate that even minor deviations from procedural fairness can influence adolescents’ trust, highlighting the importance of subtle procedural cues in shaping trust from an early age.

## Conclusion

Although prior research has established that procedural justice enhances trust in authorities, less is known about how these effects manifest during adolescence. Therefore, the present study examined whether adolescents at different developmental stages consider procedural justice aspects, such as voice, transparency, and predictability, when forming trust judgments about school and governmental authorities. Results showed that adolescents are sensitive to these procedural cues, with all examined aspects positively influencing trust. Transparency and voice had the most consistent effects, whereas predictability showed smaller but still significant impacts. These effects were largely stable across all stages of adolescence, indicating that the capacity to evaluate procedural fairness is present already in early adolescence. However, in post-communist countries with lower institutional trust, there was sporadic evidence that the influence of procedural aspects on trust increased with age, suggesting that contextual factors may also shape adolescents’ ability to evaluate authority behavior. Overall, these findings underscore that procedural factors are crucial not only for adults but also for adolescents, whose trust in institutions is still developing. By investing in transparent and inclusive governance practices, authorities can nurture trust that forms the foundation for engaged and civic-minded citizenship. Educators, policymakers, and government officials who adhere to principles of procedural justice can strengthen adolescents’ trust and foster their constructive engagement with institutions. Maintaining respectful dialogue with young people appears essential already in early adolescence.

## Supplementary Information

Below is the link to the electronic supplementary material.

Supplementary Material 1

## Data Availability

The dataset generated and analyzed during the current study is available in the Open Science Framework repository, https://osf.io/x9ajy/overview?view_only=13bb184e439543c4b775fc9e41d42a7c. The code necessary to reproduce the analyses is available in the Supplementary Material. The analyses were not preregistered.
