# Supplementary material for: Building Trust in Governmental and Educational Authorities in Adolescence: A Comparison of Early, Middle, and Late Adolescents in Four European Countries
Source: J Youth Adolesc. 2025 Dec 4;55(1):211–25. doi: 10.1007/s10964-025-02297-3 (PMC12816024; doi:10.1007/s10964-025-02297-3)
Supplement: Supplementary file 1 — Supplementary Material 1 [file 10964_2025_2297_MOESM1_ESM.docx]

**Supplementary Material - Building Trust in Governmental and Educational Authorities in Adolescence: A Comparison of Early, Middle, and Late Adolescents in Four European Countries**

**1. Vignettes and items in national languages**

| Section | Name | English | German | Italian | Czech | Serbian |
| --- | --- | --- | --- | --- | --- | --- |
| Generalized interpersonal trust | Instruction | Please read each of the following statements and rate the extent to which you believe each statement best describes your feelings about other people in general. | Bitte lies jede der folgenden Aussagen und bewerte, in welchem Ausmaß diese Deine Gefühle über andere Menschen im Allgemeinen beschreiben. | Per favore, leggi ciascuna delle seguenti dichiarazioni e valuta in che misura descrivono in generale i tuoi sentimenti verso le altre persone. | Přečtěte si prosím každé z následujících tvrzení a ohodnoťte, do jaké míry obecně popisují vaše pocity z ostatních lidí. | Molimo Vas pročitajte sledeće iskaze i ocenite u kojoj meri opisuju Vaša osećanja o drugim ljudima uopšteno. |
|  | inter_trust1 | Most people are trustworthy. | Die meisten Menschen sind vertrauenswürdig. | La maggior parte delle persone è affidabile. | Lidé jsou většinou důvěryhodní. | Većina ljudi je dostojna poverenja. |
|  | inter_trust2 | Most people are basically good and kind. | Die meisten Menschen sind grundsätzlich gut und freundlich. | La maggior parte delle persone è fondamentalmente buona e gentile. | Lidé jsou většinou v jádru dobří a laskaví. | Većina ljudi je u osnovi dobra i ljubazna. |
|  | inter_trust3 | Most people are basically honest. | Die meisten Menschen sind im Grunde ehrlich. | La maggior parte delle persone è fondamentalmente onesta. | Lidé jsou většinou v jádru čestní. | Većina ljudi je u osnovi iskrena. |
|  | inter_trust4 | Most people can be trusted. | Den meisten Menschen kann man vertrauen. | Della maggior parte delle persone ci si può fidare. | Většině lidí lze důvěřovat. | Većini ljudi se može verovati |
|  | inter_trust5 | Most of the time people are helpful. | Die meiste Zeit über sind Menschen hilfsbereit. | Il più delle volte le persone sono disponibili. | Lidé se většinu času snaží pomáhat druhým. | U većini slučajeva ljudi će rado pomoći drugima. |
|  | Instruction | Now you will read a fictional example of decision-making at school. Please read the introductory text carefully and then answer the questions that follow. When answering all questions, please keep in mind the introductory text. You can return to the text at any time when answering the questions. | Es folgt nun ein fiktives Beispiel für die Entscheidungsfindung in einer Schule. Zur Einführung in die Situation lies Dir bitte den Einführungstext aufmerksam durch und beantworte anschließend die Fragen, die auf den Text folgen. Versuche Dich bei der Beantwortung aller Fragen auf den Einführungstext zu beziehen. Du kannst bei der Beantwortung der Fragen jederzeit zum Text zurückgehen, um nochmal nachzulesen. | Seguirà un esempio di fantasia che fa riferimento a decisioni prese dalla dirigenza in una scuola. Per entrare adeguatamente nella situazione fittizia, leggi attentamente il testo introduttivo e rispondi alle domande che seguono. Cerca di rispondere a tutte le domande in relazione al testo introduttivo. Puoi sempre tornare al testo durante la risposta alle singole domande. | Nyní bude následovat smyšlený příklad rozhodování ve škole. Pro uvedení do situace si prosím nejdříve pečlivě přečtěte úvodní text a následně odpovězte na otázky, které na text navazují. Při odpovídání na všechny otázky se prosím pokuste odpovídat v návaznosti na úvodní text. K textu se můžete kdykoli při odpovídání na jednotlivé otázky vrátit. | Sada sledi izmišljeni primer donošenja odluke u školi. Molimo te da najpre pažljivo pročitaš uvodni tekst i zatim odgovoriš na pitanja koja slede. Probaj da razmišljaš o uvodnom tekstu dok odgovaraš na sva pitanja. Možeš i ponovo čitati tekst dok odgovaraš na svako pitanje. |
|  | Introduction | Imagine that you visit a school where several cases of cyberbullying using mobile phones have happened recently. Some students used their phones to mock and humiliate others. Students, teachers, and parents think that the school management has to do something because the bullying has been very serious and might happen again and again. Therefore, the school management decides to take tough steps. They decide to impose a ban on using mobile phones. Students are not allowed to use their mobile phones in classrooms under a penalty. This decision is announced to students at a joint meeting with the management. | Stelle Dir vor, Du besuchst eine Schule, an der in letzter Zeit mehrere Fälle von Cybermobbing mit Handys vorkamen. Einige Schülerinnen und Schüler nutzen ihre Handys, um andere zu verspotten und zu demütigen. Schülerinnen und Schüler, Lehrkräfte und Eltern sind der Meinung, dass die Schulleitung etwas unternehmen muss, weil das Mobbing sehr ernst war und erneut vorkommen könnte. Darum entscheidet die Schulleitung harte Schritte einzuleiten. Sie beschließt ein Handyverbot. Das Mitbringen von Handys in die Schule ist den Schülerinnen und Schülern verboten. Die Entscheidung wird den Schülerinnen und Schülern bei einem gemeinsamen Treffen mit der Schulleitung in der Turnhalle mitgeteilt. | Immaginate di visitare una scuola dove di recente si sono verificati diversi casi di cyberbullismo attraverso l’uso del cellulare. Alcuni studenti hanno usato i loro telefoni per deridere e umiliare gli altri. Studenti, insegnanti e genitori pensano che la direzione della scuola dovrebbe fare qualcosa, perché gli episodi di bullismo sono stati molto gravi e potrebbero ripetersi. Pertanto, la direzione della scuola decide di prendere provvedimenti severi. Decide di vietare l’uso dei telefoni cellulari. Agli studenti non è consentito portare a scuola i telefoni cellulari, pena una sanzione. Questa decisione viene comunicata agli studenti durante una riunione congiunta con la direzione in palestra. | Představte si, že chodíte na školu, kde se nedávno objevilo několik případů šikany přes mobilní telefony. Někteří studenti používali ve třídách své telefony, aby zesměšňovali a ponižovali druhé. Studenti, učitelé a rodiče si myslí, že by s tím vedení školy mělo něco udělat, protože šikana byla velmi závažná a může se znova opakovat. Proto se vedení školy rozhodne pro tvrdá opatření. Rozhodnou se zakázat používání mobilních telefonů. Studenti nesmí své telefony ve třídách používat, jinak budou potrestáni. Rozhodnutí je studentům oznámeno na společném setkání s vedením školy. | Zamisli da si u poseti školi u kojoj se nedavno dogodilo nekoliko slučajeva vršnjačkog nasilja putem interneta. Neki učenici su koristili svoje telefone da ismevaju i ponižavaju druge. Učenici, nastavnici i roditelji smatraju da uprava škole treba da preduzme nešto jer je vršnjačko nasilje bilo vrlo ozbiljno i može da se ponovi. Zato je uprava škole odlučila da preduzme ozbiljne korake. Odlučili su da uvedu zabranu korišćenja mobilnih telefona. Učenicima nije dozvoljeno da donesu mobilne telefone u školu, a ako to ipak učine, biće kažnjeni. Ova odluka je objavljena učenicima na zajedničkom sastanku sa upravom škole. |
|  | Voice 0 | The management decide on the measures by themselves. They are not interested in the opinions of citizens or experts and do not take them into account when making decisions. | Die Schulleitung trifft die Enscheidung über die Maßnahmen allein. Sie ist nicht an den Meinungen der Schülerinnen und Schüler interessiert. Bei der Entscheidungsfindung berücksichtigt sie die Meinungen der Schülerinnen und Schüler nicht. | I dirigenti decidono da soli le misure da adottare. Non sono interessati alle opinioni di cittadini o di esperti e non ne tengono conto quando prendono le decisioni. | Vedení rozhodne o opatřeních samo. Při rozhodování se nezajímá o názory studentů ani odborníků a nebere je v úvahu. | Školska uprava sama odlučuje o merama. Oni nisu zainteresovani za mišljenja učenika ili eksperata i ne uzimaju ih u obzir prilikom donošenja odluka. |
|  | Voice 1 | The management are interested in students’ opinions before taking a decision. When deciding, they collect students’ opinions using anonymous online polls or pieces of paper and consider students’ opinions carefully. | Die Schulleitung ist an den Meinungen der Schülerinnen und Schüler interessiert, bevor sie Entscheidungen trifft. Bei der Entscheidungsfindung holt sie die Meinungen der Schülerinnen und Schüler mittels anonymer Online- oder Papier-Umfragen ein und berücksichtigt die Meinungen der Schülerinnen und Schüler sorgfältig. | La direzione è interessata alle opinioni degli studenti prima di prendere una decisione. Al momento di prendere una decisione, raccoglie le opinioni degli studenti tramite sondaggi online anonimi o tramite moduli cartacei anonimi e considera le risposte con attenzione. | Dříve než rozhodne, vedení školy se aktivně zajímá o názory studentů. Při rozhodování proto udělá mezi studenty anonymní anketu, kdy studenti mohli napsat svůj názor online nebo na kousek papíru. Vedení pak názory studentů při zavádění konkrétních opatření pečlivě zváží. | Školsku upravu interesuje mišljenje učenika pre neko što donese odluku. Kada uprava odlučuje, prikuplja mišljenja koristeći anonimne upitnike popunjene onlajn ili na papiru i pažljivo uzima u obzir mišljenja učenika. |
|  | Voice 2 | The management are interested in expert opinions before they take a decision. When deciding, they approach academic experts in cyberbullying, youth workers, and exeprienced teachers. The ban on using phones is thoroughly consulted with these experts. | Die Schulleitung ist an Expertenmeinungen interessiert, bevor sie Entscheidungen trifft. Bei der Entscheidungsfindung wendet sie sich an wissenschaftliche Fachleute für Cybermobbing, an Sozialarbeiterinnen und Sozialarbeiter und an erfahrene Lehrkräfte. Das Handyverbot wird mit diesen Expertinnen und Experten sorgfältig beraten. | La direzione è interessata alle opinioni di alcuni esperti prima di prendere una decisione. Al momento di prendere una decisione, la direzione si rivolge a esperti universitari di cyberbullismo, operatori giovanili ed insegnanti con esperienza. La decisione di vietare i telefoni viene discussa in modo approfondito con questi esperti. | Dříve než rozhodne, vedení školy se zajímá o názory odborníků. Při rozhodování proto osloví experty na kyberšikanu z univerzity, pracovníky s mládeží a zkušené učitele. Zákaz telefonů je důkladně konzultován s těmito experty. | Školsku upravu interesuje mišljenje stručnjaka pre nego što donese odluku. Pre odluke se obraća naučnicima koji se bave vršnjačkim nasiljem putem interneta, edukatorima i iskusnim nastavnicima. O zabrani korišćenja mobilnih telefona se temeljno savetuje sa ovim stručnjacima. |
|  | Transp 0 | The management do not explain their decision in any way. They present to the students only their final decision but not the specific reasons based on which the decision has been made. They also do not explain why such a solution has been chosen over other options. | Die Schulleitung erklärt ihre Entscheidungen in keiner Weise. Die Schulleitung präsentiert den Schülerinnen und Schülern nur ihre finale Entscheidung, aber nicht die spezifischen Gründe für diese Entscheidung. Sie erklärt zudem nicht, warum sie die aktuelle Lösung gegenüber anderen Möglichkeiten bevorzugt. | La direzione non spiega in alcun modo la propria decisione. La direzione si limita a presentare agli studenti la decisione finale, ma non le ragioni specifiche in base alle quali è stata presa. Inoltre, la direzione non spiega il motivo per cui è stata scelta tale soluzione rispetto ad altre opzioni. | Vedení své rozhodnutí nijak nevysvětluje. Studentům představí pouze své konečné rozhodnutí, ne však konkrétní důvody, na základě kterých bylo rozhodnuto. Rovněž nevysvětlí, proč bylo vybráno takové řešení před jinými možnostmi. | Školska uprava ni na koji način ne objašnjava svoje odluke. Predstavljaju učenicima samo svoju konačnu odluku, ali ne i konkretne razloge na osnovu kojih je odluka doneta. Takođe, ne objašnjavaju zbog čega je takvo rešenje odabrano u odnosu na druge opcije. |
|  | Transp 1 | The management do their best to explain the decision carefully. The management shed light on all reasons, arguments, and facts that have been considered. They make clear why they prefer the current solution over other possibilities. | Die Schulleitung tut ihr Bestes, um ihre Entscheidungen sorgfältig zu erklären. Die Schulleitung beleuchtet alle Gründe, Argumente und Fakten, die berücksichtigt wurden. Sie macht deutlich, warum sie die aktuelle Lösung gegenüber anderen Möglichkeiten bevorzugt. | La direzione fa del proprio meglio per spiegare la decisione in modo accurato. La direzione fa luce su tutte le ragioni, gli argomenti e i fatti che sono stati presi in considerazione. Chiarisce perché preferisce la soluzione attuale rispetto ad altre possibilità. | Vedení školy se pokusí co nejpečlivěji vysvětlit své rozhodnutí. Objasní všechny důvody, argumenty a fakta, která vzalo při rozhodování do úvahy. Vysvětlí, proč dávají přednost vybranému řešení před jinými možnostmi. | Školska uprava čini sve što može da pažljivo objasni odluku. Razjašnjavaju se svi razlozi, argumenti i činjenice koje su uzete u obzir pri donošenju odluke. Uprava pojašnjava zašto se preferira ovo rešenje nad drugim mogućnostima. |
|  | Predict 0 | The management present their decision saying that it will probably change continuously. Students thus do not know a clear plan of what measures will be taken and when. It is also difficult for students to prepare for the measures in advance. | Die Schulleitung stellt ihre Entscheidung mit dem Hinweis vor, dass sie sich wahrscheinlich ständig ändern wird. Die Schülerinnen und Schülern haben also keinen klaren Plan davon, wann welche Maßnahmen ergriffen werden. Es ist schwierig für die Schülerinnen und Schüler, sich im Voraus auf die Maßnahmen vorzubereiten. | La direzione presenta la propria decisione dicendo che tale decisione sarà probabilmente soggetta a ulteriori cambiamenti. Gli studenti non conoscono quindi un piano chiaro di quali misure saranno adottate e quando. È anche difficile per gli studenti prepararsi alle misure in anticipo. | Vedení představí své rozhodnutí s tím, že se nejspíš bude průběžně měnit. Studenti tak neznají jasný plán, kdy bude zákaz zaveden, jak bude kontrolováno jeho dodržování a jak budou vypadat tresty. Rovněž je pro ně obtížné se na zákaz dopředu připravit. | Uprava škole predstavlja svoju odluku navodeći da će se ona menjati u hodu. Stoga, učenici ne znaju jasan plan mera koje će biti preduzete i kada. Takođe, učenicima je teško da se unapred pripreme za te mere. |
|  | Predict 1 | The management wants to give students some certainty. They tell students a clear plan on when the ban will be imposed, how the ban will be monitored, and what the penalty will look like. This helps students to prepare in advance. | Die Schulleitung möchte den Schülerinnen und Schülern eine gewisse Sicherheit geben. Sie teilt den Schülerinnen und Schülern einen klaren Plan mit, wann das Verbot verhängt wird, wie es überwacht wird und wie die Strafe aussehen wird. Dies hilft den Schülerinnen und Schülern, sich im Voraus vorzubereiten. | La direzione vuole dare agli studenti una certa sicurezza, indicando loro un piano chiaro su quando verrà imposto il divieto, su come verrà monitorato e su quale sarà la sanzione. Ciò aiuta gli studenti a prepararsi in anticipo. | Vedení školy chce dát studentům nějakou jistotu. Proto jim přestaví jasný plán toho, kdy bude zákaz zaveden, jak bude kontrolováno jeho dodržování a jak budou vypadat tresty. Pomůže tím studentům, aby se mohli na zákaz dopředu připravit. | Školska uprava želi da pruži učenicima neku sigurnost. Predstavlja im jasan plan kada će se zabrana uvesti, kako će se kontrolisati, i kakve kazne će se uvesti. Ovo pomaže učenicima da se pripreme na vreme. |
| Trust in school management | Instruction 1 | People may differ in their perception of such a situation. The decision itself can be important, but also how the school management behave to the students. How would you personally feel if the school management behaved in the manner described, and what would you think of such school management? | Menschen können eine solche Situation unterschiedlich wahrnehmen. Die Entscheidung selbst kann wichtig sein, aber auch die Art und Weise, wie die Schülerinnen und Schüler behandelt werden. Wie würdest Du dich persönlich fühlen, wenn sich die Schulleitung in der beschriebenen Weise verhalten würde, und was würdest Du von der Schulleitung halten? | Le persone possono avere una percezione diversa di questa situazione. La decisione in sé può essere importante, ma anche il comportamento della direzione scolastica nei confronti degli studenti. Come vi sentireste personalmente se la direzione della scuola si comportasse nel modo descritto e cosa pensereste di questa gestione? | Různí lidé mohou takovouto situaci vnímat odlišně. Důležité může být samotné rozhodnutí, ale i to, jak vedení školy jednalo se studenty. Jak byste se vy osobně cítil/a, kdyby se vedení školy zachovalo popsaným způsobem, a co byste si o takovém vedení školy myslel/a? | Ljudi na različite načine doživljavaju ovakve situacije. Sama odluka je važna, ali to važi i za način na koji uprava škole razgovara sa učenicima. Kako bi se ti lično osećao/la kada bi se uprava ponašala na opisan način, i šta bi mislio/la o njoj? |
|  | Instruction 2 | Based on the described experience with how the school management behaved to the students … | Ausgehend von der beschriebenen Erfahrung, wie die Schulleitung die Schülerinnen und Schüler behandelt hat, ... | Sulla base della situazione descritta ed in base al comportamento della direzione scolastica nei confronti degli studenti ... | Na základě popsané zkušenosti s tím, jak jednalo vedení školy se studenty, … | Na osnovu prethodno opisanog iskustva o tome kako je uprava škole tretirala učenike, molimo te da odrediš kako bi se osećao/la kad bi doživeo/la ovakav odgovor uprave na situaciju. |
|  | vman_t1 | I would be comfortable with being vulnerable to the judgement of such management. | ... Ich würde mich damit wohlfühlen, mich dem Urteil der Schulleitung auszusetzen. | ... Mi sentirei a mio agio nell’essere soggetto alla valutazione della direzione. | ... bych se klidně svěřil/a do rukou takového vedení školy a spoléhal/a na jejich úsudek. | ... Bilo bi mi ugodno da budem izložen/a procenjivanju od strane takve školske uprave. |
|  | vman_t2 | I would be open to letting such management make more decisions about issues that are important to me. | ... Ich wäre offen dafür, die Schulleitung mehr Entscheidungen über Themen treffen zu lassen, die mir wichtig sind. | ... Sarei disposto a lasciare che la direzione prenda più decisioni su questioni per me importanti. | ... bych byl/a ochotný/á nechat takové vedení školy rozhodovat i o dalších věcech, které jsou pro mě důležité. | ... Bio/la bih otvoren/a da pustim takvu upravu da donese više odluka o pitanjima koja su mi važna. |
|  | vman_t3 | I would be comfortable with letting such management make more decisions that may affect my future. | ... Ich wäre damit einverstanden, die Schulleitung mehr Entscheidungen treffen zu lassen, die sich auf meine Zukunft auswirken könnten. | ... Sarei a mio agio nel lasciare che la direzione prenda più decisioni che possono influenzare il mio futuro. | ... bych klidně nechal/a takové vedení školy udělat i další rozhodnutí, která by mohla ovlivnit mou budoucnost. | ... Bilo bi mi ugodno da pustim takvu upravu da donese više odluka koje mogu da utiču na moju budućnost. |
|  | vman_t4 | I would expect that letting such management make decisions won’t harm me. | ... Ich würde erwarten, dass es mir nicht schadet, die Schulleitung Entscheidungen treffen zu lassen. | ... Mi aspetto che lasciare che la direzione prenda decisioni non mi danneggi. | ... bych očekával/a, že když nechám takové vedení školy rozhodovat, tak mi to nijak neublíží. | ... Očekivao/la bih da me prepuštanje odluke takvoj upravi ne bi oštetilo. |
| Vignette government | Instruction | Now we will be interested in your views on state-level decision-making. We will use a fictional example of another pandemic, which can be related to the adoption of various anti-pandemic measures. Again, please read the introductory text carefully and then answer the following questions. | Uns interessiert nun, was Du über verschiedenen Arten der Entscheidungsfindung auf Regierungsebene denkst. Wir verwenden ein fiktives Beispiel für eine weitere Pandemie, mit der die Einführung verschiedener Schutzmaßnahmen verbunden sein kann. Bitte lies Dir den einleitenden Text sorgfältig durch und beantworte dann die Fragen, die auf den einleitenden Text folgen. | Ora ci interessa conoscere le tue opinioni sui diversi modi in cui lo stato prende decisioni. Per questo, useremo un esempio immaginario di un'altra pandemia, che potrebbe comportare l'attuazione di diverse misure. Per favore, rileggi attentamente il testo introduttivo e poi rispondi alle domande che seguono il testo introduttivo. | Nyní nás bude zajímat, jaké jsou vaše názory na různé způsoby rozhodování státu. Využijeme k tomu smyšlený příklad další pandemie, s níž může souviset zavádění různých opatření. Opět si prosím pečlivě přečtěte úvodní text a následně odpovězte na otázky, které na úvodní text navazují. | Sada nas interesuju Vaši stavovi o donošenju odluka na nivou države. Koristićemo fiktivni primer neke druge pandemije koji može biti povezan sa usvajanjem raznih anti-pandemijskih mera. Još jednom Vas molimo da pažljivo pročitate uvodni tekst i potom odgovorite na pitanja koja slede zamišljajući tu situaciju. |
|  | Introduction | Imagine that another pandemic of infectious disease is coming to our country. It can be a strong flu or another variant of covid. The government have to take action. It seems that the mandatory testing and wearing of facemasks in some places are the most effective moves at this moment. Therefore, the government assemble and decide on specific places where facemasks will be mandatory. They also determine in which situations people have to take tests. All measures are first announced in the evening television news by the health minister. | Stelle Dir vor, dass eine weitere Pandemie mit einer Infektionskrankheit dein Land erreicht. Es kann eine starke Grippe oder eine andere Covid-Variante sein. Die Regierung muss handeln. Verpflichtende Tests und das Tragen von Gesichtsmasken an bestimmten Orten scheinen im Moment die effektivsten Maßnahmen zu sein. Aus diesem Grund trifft sich die Regierung und entscheidet über bestimmte Orte, an denen das Tragen von Gesichtsmasken vorgeschrieben wird. Sie bestimmt auch, in welchen Situationen Personen getestet werden müssen. Alle Maßnahmen werden das erste Mal in den Abendnachrichten vom Gesundheitsminister bekanntgegeben. | Immaginate che una nuova pandemia legata ad una malattia infettiva stia arrivando nel nostro Paese. Può trattarsi di una forte influenza o di un’altra variante di coronavirus. Il governo deve intervenire. Sembra che i test obbligatori e l’uso di mascherine in alcuni luoghi rappresentino le mosse più efficaci per far fronte alla pandemia. Pertanto, il governo si riunisce e decide i luoghi specifici in cui sarà obbligatorio indossare le mascherine. Il governo stabilisce anche in quali situazioni le persone devono sottoporsi ai test. Tutte le misure vengono annunciate per la prima volta dal ministro della Sanità durante il notiziario televisivo serale. | Představte si, že se v naší zemi objevila další pandemie nakažlivé nemoci. Může to být silná chřipka nebo další varianta covidu. Vláda se rozhodla jednat. Zdá se, že nejúčinnější opatření jsou v tuto chvíli povinné testování a nošení roušek na některých místech. Vláda se proto sejde a rozhodne, kde přesně bude nošení roušek povinné. Rovněž rozhodne, v jakých situacích se lidé budou muset otestovat. Všechna opatření poprvé představí ministr zdravotnictví ve večerních televizních zprávách. | Zamislite da stiže još jedna pandemija zarazne bolesti u našu zemlju. To može biti jak oblik gripa ili još jedna varijanta kovida. Vlada treba da deluje. Izgleda da su se obavezno testiranje i nošenje maski na nekim mestima pokazali kao trenutno najefikasnije mere. Zato se vlada sastaje i odlučuje na kojim tačno mestima će biti obavezno nošenje maski. Takođe određuje u kojim situacijama ljudi moraju da se testiraju. Sve mere prvo objavljuje ministar/ka zdravlja u večernjim televizijskim vestima. |
|  | Voice 0 | The government decide on the measures by themselves. They are not interested in the opinions of citizens or experts and do not take them into account when making decisions. | Die Regierung trifft die Enscheidung über die Maßnahmen allein. Sie ist nicht an den Meinungen der Bürgerinnen und Bürger oder Expertinnen und Experten interessiert und berücksichtigt diese bei der Entscheidungsfindung nicht. | Il governo decide le misure da solo. Non è interessato alle opinioni dei cittadini o degli esperti e non le prende in considerazione quando prende le decisioni. | Vláda rozhodne o opatřeních sama. Při rozhodování se nezajímá o názory občanů ani odborníků a nebere je v úvahu. | Vlada sama odlučuje o merama. Oni nisu zainteresovani za mišljenja građana ili eksperata i ne uzimaju ih u obzir prilikom donošenja odluka. |
|  | Voice 1 | The government are interested in citizens’ opinions before taking a decision. When deciding, they carefully consider all public opinion polls on this issue and petitions from ordinary citizens. | Die Regierung interessiert sich für die Meinungen der Bürgerinnen und Bürger, bevor sie eine Entscheidung trifft. Bei ihrer Entscheidung berücksichtigt sie sorgfältig alle öffentlichen Meinungsumfragen zu dem Thema und Petitionen der Bürgerinnen und Bürgern. | Il governo si preoccupa di considerare le opinioni dei cittadini prima di prendere una decisione. Al momento di decidere, considera attentamente tutti i sondaggi di opinione su questo tema, nonché petizioni avanzate da comuni cittadini. | Dříve než rozhodne, vláda se aktivně zajímá o názory občanů. Při rozhodování se proto pečlivě zajímá o všechny průzkumy veřejného mínění na toto téma spolu s peticemi a dalšími dostupnými informacemi od obyčejných občanů. Vláda pak názory občanů při zavádění konkrétních opatření pečlivě zváží. | Vlada je zainteresovana za mišljenje građana pre nego što donese odluke. Kada odlučuje, vlada pažljivo uzima u obzir sva istraživanja javnog mnjenja o datom pitanju, kao i peticije običnih građana. |
|  | Voice 2 | The government are interested in expert opinions before they take a decision. When deciding, they assemble a board of experts in epidemiology, virology, sociology, economy and related disciplines. All measurers are thoroughly consulted with these experts. | Die Regierung interessiert sich für Expertenmeinungen, bevor sie eine Entscheidung trifft. Vor der Entscheidung versammelt sie ein Expertengremium aus den Bereichen Epidemiologie, Virologie, Soziologie, Wirtschaft und verwandten Disziplinen. Alle Maßnahmen werden gründlich mit diesen Expertinnen und Experten besprochen. | Il governo è interessato a considerare opinioni offerte da esperti prima di prendere una decisione. Quando il governo decide, riunisce un comitato di esperti in epidemiologia, virologia, sociologia, economia e discipline correlate. Tutti i provvedimenti vengono discussi in modo approfondito con questi esperti. | Dříve než rozhodne, vláda se zajímá o názory odborníků. Při rozhodování se proto sejde tým expertů na epidemiologii, virologii, sociologii, ekonomii a další obory. Všechna opatření jsou s těmito experty důkladně konzultována. | Vlada je zainteresovana za mišljenje stručnjaka pre nego što donese odluke. Kada odlučuje, vlada saziva komisiju eksperata u poljima epidemiologije, virologije, sociologije, ekonomije i povezanih disciplina. O svim merama se pažljivo konsultuje sa ovim stručnjacima. |
|  | Transp 0 | The government do not explain their decision in any way. They present to the citizens only their final decision but not the specific reasons based on which the decision has been made. They also does not explain why such a solution has been chosen over other options. | Die Regierung erklärt ihre Entscheidungen in keiner Weise. Sie präsentiert den Bürgerinnen und Bürgern nur ihre finale Entscheidung, aber nicht die spezifischen Gründe für diese Entscheidung. Sie erklärt zudem nicht, warum sie die aktuelle Lösung gegenüber anderen Möglichkeiten bevorzugt. | Il governo non spiega in alcun modo la propria decisione. Presenta ai cittadini solo la sua decisione finale, ma non le ragioni specifiche per cui è stata presa. Inoltre, non spiega perché sia stata scelta tale soluzione rispetto ad altre opzioni. | Vláda své rozhodnutí nijak nevysvětluje. Občanům představí pouze své konečné rozhodnutí, ne však konkrétní důvody, na základě kterých bylo rozhodnuto. Rovněž nevysvětlí, proč bylo vybráno takové řešení před jinými možnostmi. | Vlada ni na koji način ne objašnjava svoje odluke. Predstavljaju građanima samo svoju konačnu odluku, ali ne i konkretne razloge na osnovu kojih je odluka doneta. Takođe, ne objašnjavaju zbog čega je takvo rešenje odabrano u odnosu na druge opcije. |
|  | Transp 1 | The government do their best to explain the decision carefully. The government shed light on all reasons, arguments, and facts that have been considered. They make clear why they prefer the current solution over other possibilities. | Die Regierung tut ihr Bestes, um ihre Entscheidung sorgfältig zu erklären. Die Regierung legt alle Gründe, Argumente und Fakten dar, die berücksichtigt wurden. Sie macht deutlich, warum sie die derzeitige Lösung gegenüber anderen Möglichkeiten vorzieht. | Il governo fa del suo meglio per spiegare accuratamente le decisioni. Il governo fa luce su tutte le ragioni, gli argomenti e i fatti che sono stati presi in considerazione. Chiarisce perché preferisce una soluzione rispetto ad altre possibilità. | Vláda se pokusí co nejpečlivěji vysvětlit své rozhodnutí. Objasní všechny důvody, argumenty a fakta, která byla vzata při rozhodování do úvahy. Současně jasně vysvětlí, proč dávají přednost vybranému řešení před jinými možnostmi. | Vlada čini sve što može da pažljivo objasni odluku. Vlada rasvetljava sve razloge, argumente i činjenice koje su uzete u obzir pri donošenju odluke. Pojašnjava zašto se preferira ovo rešenje u odnosu na druge mogućnosti. |
|  | Predict 0 | The government present their decision saying that it will probably change continuously. People thus do not know a clear plan of what measures will be taken and when. It is also difficult for people to prepare for the measures in advance. | Die Regierung stellt ihre Entscheidung mit dem Hinweis vor, dass sie sich wahrscheinlich ständig ändern wird. Die Bürgerinnen und Bürger haben also keinen klaren Plan davon, wann welche Maßnahmen ergriffen werden. Es ist schwierig für die Bürgerinnen und Bürger, sich im Voraus auf die Maßnahmen vorzubereiten. | Il governo presenta la sua decisione dicendo che probabilmente sarà soggetta ad ulteriori cambiamenti. Le persone non conoscono quindi un piano chiaro di quali misure saranno adottate e quando. È anche difficile per le persone prepararsi alle misure in anticipo. | Vláda představí své rozhodnutí s tím, že se nejspíš bude průběžně měnit. Lidé tak neznají jasný plán, jaká opatření budou přijata a kdy. Rovněž je pro ně obtížné se na opatření dopředu připravit. | Vlada predstavlja svoju odluku navodeći da će se ona menjati u hodu. Stoga, građani ne znaju jasan plan mera koje će biti preduzete i kada. Takođe, građanima je teško da se unapred pripreme za te mere. |
|  | Predict 1 | Even though the situation can suddenly change, the government want to give people some certainty. They present people with a clear plan, showing them what measures will be taken and when. This helps people to prepare in advance. | Jeder weiß, dass sich die Situation plötzlich ändern kann, aber die Regierung will den Menschen eine gewisse Sicherheit geben. Sie präsentiert den Menschen einen klaren Plan, der ihnen zeigt, welche Maßnahmen wann ergriffen werden. Dies hilft den Menschen, sich im Voraus vorzubereiten. | Tutti sanno che la situazione può cambiare improvvisamente, ma il governo vuole dare ai cittadini una certa sicurezza. Presenta ai cittadini un piano chiaro, indicando quali misure saranno adottate e quando saranno adottate. Questo aiuta le persone a prepararsi in anticipo. | I když se situace může náhle změnit, vláda chce i přesto dát občanům určitou jistotu. Proto představí lidem jasný plán toho, jaká opatření budou přijata a kdy. Pomůže tím lidem, aby se na opatření mohli připravit dopředu. | Iako se situacija može iznenada promeniti, vlada želi da građanima pruži neku sigurnost. Predstavlja se jasan plan, objašnjava se koje mere će biti preduzete i kada. Ovo pomaže da se građani na vreme pripreme. |
| Trust in government | Instruction 1 | Again, people may differ in their perception of such a situation. The decision itself can be important, but also how the government behave to the citizens. How would you personally feel if the government behaved in the manner described, and what would you think of such government? | Auch hier gilt, dass Menschen eine solche Situation unterschiedlich wahrnehmen können. Die Entscheidung selbst kann wichtig sein, aber auch, wie die Regierung mit den Bürgerinnen und Bürgern umgegangen ist. Wie würdest Du Dich persönlich fühlen, wenn sich die Regierung in der beschriebenen Weise verhalten würde, und was würdest Du über die Regierung denken? | Anche qui diverse persone potrebbero percepire questa situazione in modo diverso. La decisione in sé potrebbe essere importante, ma anche come il governo ha trattato i cittadini. Come ti sentiresti personalmente se il governo si fosse comportato in questo modo descritto e cosa penseresti di questo governo? | Opět i zde mohou různí lidé takovouto situaci vnímat odlišně. Důležité může být samotné rozhodnutí, ale i to, jak vláda jednala s občany. Jak byste se vy osobně cítil/a, kdyby se vláda zachovala popsaným způsobem, a co byste si o této vládě myslel/a? | Ponovo, ljudi mogu različito da doživljavaju ovakve situacije. Sama odluka je važna, ali to važi i za način na koji vlada stupa u odnos sa građanima. Kako biste se Vi lično osećali kada bi se vlada ponašala na opisan način, i šta biste mislili o njoj? |
|  | Instruction 2 | Based on the described experience with how the government behaved to the citizens … | Ausgehend von der oben beschriebenen Erfahrung, wie die Regierung die Bürgerinnen und Bürger behandelt hat, ... | Sulla base dell'esperienza descritta di come il governo ha trattato i cittadini, ... | Na základě popsané zkušenosti s tím, jak vláda výše jednala s občany, … | Na osnovu prethodno opisanog iskustva o tome kako je vlada tretirala građane, molimo Vas odredite kako biste se osećali kad biste doživeli ovakav odgovor vlade na situaciju. ... |
|  | vgov_t1 | I would be comfortable with being vulnerable to the judgement of such government. | ... Ich würde mich damit wohlfühlen, mich dem Urteil der Regierung auszusetzen. | ... Mi sentirei a mio agio nell’essere soggetto alle decisioni del governo. | ... bych se klidně svěřil/a do rukou takové vlády a spoléhal/a na její úsudek. | ... Bilo bi mi ugodno da budem izložen/a procenjivanju od strane takve vlade. |
|  | vgov_t2 | I would be open to letting such government make more decisions about issues that are important to me. | ... Ich wäre offen dafür, die Regierung mehr Entscheidungen über Themen treffen zu lassen, die mir wichtig sind. | ... Sarei disposto a lasciare che il governo prenda più decisioni su questioni per me importanti. | ... bych byl/a ochotný/á nechat takovou vládu rozhodovat i o dalších věcech, které jsou pro mě důležité. | ... Bio/la bih otvoren/a da pustim takvu vladu da donese više odluka o pitanjima koja su mi važna. |
|  | vgov_t3 | I would be comfortable with letting such government make more decisions that may affect my future. | ... Ich wäre damit einverstanden, die Regierung mehr Entscheidungen treffen zu lassen, die sich auf meine Zukunft auswirken könnten. | ... Sarei a mio agio nel lasciare che il governo prenda più decisioni che possono influenzare il mio futuro. | ... bych klidně nechal/a takovou vládu udělat i další rozhodnutí, která by mohla ovlivnit mou budoucnost. | ... Bilo bi mi ugodno da pustim takvu vladu da donese više odluka koje mogu da utiču na moju budućnost. |
|  | vgov_t4 | I would expect that letting such government make decisions won’t harm me. | ... Ich würde erwarten, dass es mir nicht schadet, die Regierung Entscheidungen treffen zu lassen. | ... Mi aspetto che lasciare che il governo prenda decisioni non mi danneggi. | ... bych očekával/a, že když nechám takovou vládu rozhodovat, tak mi to nijak neublíží. | ... Očekivao/la bih da me prepuštanje odluke takvoj vladi ne bi oštetilo. |

**2. Power analysis**

***R code:***

library(Superpower)

# age group (3 levels) X voice (3 levels)

# 11-12: no effects

# 14-15: small (0.2SD) effects of citizen and expert voice

# 18-19: large (0.8SD) effects of citizen and expert voice

design_result <- ANOVA_design( design = "3b*3b", n = 66, mu = c(0, 0, 0, 0, 0.2, 0.2, 0.0, 0.8, 0.8), sd = 1) plot_power(design_result, min_n = 10, max_n = 100, desired_power = 80, plot = TRUE)

# age group (3 levels) X transparency or predictability (2 levels)

# 11-12: no effect

# 14-15: a small (0.2SD) effect

# 18-19: a large (0.8SD) effect

design_result <- ANOVA_design( design = "3b*2b", n = 100, mu = c(0, 0, 0, 0.2, 0, 0.8), sd = 1) plot_power(design_result, min_n = 10, max_n = 100, desired_power = 80, plot = TRUE)

***Results***

Number of participants per cell required to achieve 80% power

|  | Voice as an experimental condition | Transparency or predictability as an experimental condition |
| --- | --- | --- |
| Age group | 22 | 57 |
| Experimental condition | 44 | 48 |
| Age * Experimental condition | 53 | 57 |

**3. Full factorial ANCOVA results**

***Trust in school management***

Model 1 (no interactions)

|  | Germany | | | |  | Italy | | | |  | Czechia | | | |  | Serbia | | | |
| --- | --- | --- | --- | --- | --- | --- | --- | --- | --- | --- | --- | --- | --- | --- | --- | --- | --- | --- | --- |
|  | F | df | p | η^2^ |  | F | df | p | η^2^ |  | F | df | p | η^2^ |  | F | df | p | η^2^ |
| Voice | 10.30 | 2, 588 | 0.00 | 0.03 |  | 4.88 | 2, 410 | 0.01 | 0.02 |  | 14.38 | 2, 706 | 0.00 | 0.04 |  | 11.84 | 2, 606 | 0.00 | 0.04 |
| Transparency in rationale | 20.90 | 1, 588 | 0.00 | 0.03 |  | 8.41 | 1, 410 | 0.00 | 0.02 |  | 27.53 | 1, 706 | 0.00 | 0.04 |  | 15.28 | 1, 606 | 0.00 | 0.02 |
| Predictable framework | 3.61 | 1, 588 | 0.06 | 0.01 |  | 6.09 | 1, 410 | 0.01 | 0.01 |  | 9.15 | 1, 706 | 0.00 | 0.01 |  | 15.67 | 1, 606 | 0.00 | 0.03 |
| Age group | 13.76 | 2, 588 | 0.00 | 0.04 |  | 4.63 | 2, 410 | 0.01 | 0.02 |  | 10.39 | 2, 706 | 0.00 | 0.03 |  | 19.85 | 2, 606 | 0.00 | 0.06 |
| Gender | 13.14 | 1, 588 | 0.00 | 0.02 |  | 56.64 | 1, 410 | 0.00 | 0.12 |  | 11.63 | 1, 706 | 0.00 | 0.02 |  | 35.15 | 1, 606 | 0.00 | 0.05 |
| Generalized interpersonal trust | 3.10 | 3, 588 | 0.03 | 0.02 |  | 1.66 | 1, 410 | 0.20 | 0.00 |  | 0.82 | 3, 706 | 0.48 | 0.00 |  | 5.32 | 2, 606 | 0.01 | 0.02 |
| N | 599 |  |  |  |  | 419 |  |  |  |  | 717 |  |  |  |  | 616 |  |  |  |

Model 2 (with interactions)

|  | Germany | | | |  | Italy | | | |  | Czechia | | | |  | Serbia | | | |
| --- | --- | --- | --- | --- | --- | --- | --- | --- | --- | --- | --- | --- | --- | --- | --- | --- | --- | --- | --- |
|  | F | df | p | η^2^ |  | F | df | p | η^2^ |  | F | df | p | η^2^ |  | F | df | p | η^2^ |
| Voice | 9.60 | 2, 580 | 0.00 | 0.03 |  | 5.03 | 2, 402 | 0.01 | 0.02 |  | 13.53 | 2, 698 | 0.00 | 0.04 |  | 11.70 | 2, 598 | 0.00 | 0.04 |
| Transparency in rationale | 20.62 | 1, 580 | 0.00 | 0.03 |  | 7.70 | 1, 402 | 0.01 | 0.02 |  | 24.65 | 1, 698 | 0.00 | 0.03 |  | 14.46 | 1, 598 | 0.00 | 0.02 |
| Predictable framework | 3.37 | 1, 580 | 0.07 | 0.01 |  | 6.98 | 1, 402 | 0.01 | 0.02 |  | 9.82 | 1, 698 | 0.00 | 0.01 |  | 14.65 | 1, 598 | 0.00 | 0.02 |
| Age group | 12.78 | 2, 580 | 0.00 | 0.04 |  | 4.34 | 2, 402 | 0.01 | 0.02 |  | 10.37 | 2, 698 | 0.00 | 0.03 |  | 19.46 | 2, 598 | 0.00 | 0.06 |
| Gender | 12.95 | 1, 580 | 0.00 | 0.02 |  | 54.13 | 1, 402 | 0.00 | 0.12 |  | 11.37 | 1, 698 | 0.00 | 0.02 |  | 34.84 | 1, 598 | 0.00 | 0.06 |
| Generalized interpersonal trust | 3.23 | 3, 580 | 0.02 | 0.02 |  | 2.31 | 1, 402 | 0.13 | 0.01 |  | 0.96 | 3, 698 | 0.41 | 0.00 |  | 5.45 | 2, 598 | 0.00 | 0.02 |
| Voice * Age group | 1.07 | 4, 580 | 0.37 | 0.01 |  | 0.77 | 4, 402 | 0.54 | 0.01 |  | 1.15 | 4, 698 | 0.33 | 0.01 |  | 0.27 | 4, 598 | 0.90 | 0.00 |
| Transparency in rationale * Age group | 0.09 | 2, 580 | 0.91 | 0.00 |  | 3.60 | 2, 402 | 0.03 | 0.02 |  | 3.85 | 2, 698 | 0.02 | 0.01 |  | 3.13 | 2, 598 | 0.04 | 0.01 |
| Predictable framework * Age group | 1.74 | 2, 580 | 0.18 | 0.01 |  | 0.19 | 2, 402 | 0.83 | 0.00 |  | 0.99 | 2, 698 | 0.37 | 0.00 |  | 2.36 | 2, 598 | 0.10 | 0.01 |
| N | 599 |  |  |  |  | 419 |  |  |  |  | 717 |  |  |  |  | 616 |  |  |  |

***Trust in government***

Model 1 (no interactions)

|  | Germany | | | |  | Italy | | | |  | Czechia | | | |  | Serbia | | | |
| --- | --- | --- | --- | --- | --- | --- | --- | --- | --- | --- | --- | --- | --- | --- | --- | --- | --- | --- | --- |
|  | F | df | p | η^2^ |  | F | df | p | η^2^ |  | F | df | p | η^2^ |  | F | df | p | η^2^ |
| Voice | 13.25 | 2, 583 | 0.00 | 0.04 |  | 5.98 | 2, 411 | 0.00 | 0.03 |  | 30.30 | 2, 706 | 0.00 | 0.08 |  | 10.20 | 2, 598 | 0.00 | 0.03 |
| Transparency in rationale | 19.16 | 1, 583 | 0.00 | 0.03 |  | 7.58 | 1, 411 | 0.01 | 0.02 |  | 59.72 | 1, 706 | 0.00 | 0.08 |  | 7.05 | 1, 598 | 0.01 | 0.01 |
| Predictable framework | 11.87 | 1, 583 | 0.00 | 0.02 |  | 1.86 | 1, 411 | 0.17 | 0.00 |  | 14.02 | 1, 706 | 0.00 | 0.02 |  | 5.83 | 1, 598 | 0.02 | 0.01 |
| Age group | 3.38 | 2, 583 | 0.03 | 0.01 |  | 1.15 | 2, 411 | 0.32 | 0.01 |  | 0.33 | 2, 706 | 0.72 | 0.00 |  | 19.50 | 2, 598 | 0.00 | 0.06 |
| Gender | 0.52 | 3, 583 | 0.67 | 0.00 |  | 2.20 | 1, 411 | 0.14 | 0.01 |  | 0.70 | 3, 706 | 0.55 | 0.00 |  | 2.52 | 2, 598 | 0.08 | 0.01 |
| Generalized interpersonal trust | 37.10 | 1, 583 | 0.00 | 0.06 |  | 64.37 | 1, 411 | 0.00 | 0.14 |  | 11.45 | 1, 706 | 0.00 | 0.02 |  | 20.52 | 1, 598 | 0.00 | 0.03 |
| N | 594 |  |  |  |  | 420 |  |  |  |  | 717 |  |  |  |  | 608 |  |  |  |

Model 2 (with interactions)

|  | Germany | | | |  | Italy | | | |  | Czechia | | | |  | Serbia | | | |
| --- | --- | --- | --- | --- | --- | --- | --- | --- | --- | --- | --- | --- | --- | --- | --- | --- | --- | --- | --- |
|  | F | df | p | η^2^ |  | F | df | p | η^2^ |  | F | df | p | η^2^ |  | F | df | p | η^2^ |
| Voice | 13.85 | 2, 575 | 0.00 | 0.05 |  | 5.58 | 2, 403 | 0.00 | 0.03 |  | 28.79 | 2, 698 | 0.00 | 0.08 |  | 10.45 | 2, 590 | 0.00 | 0.03 |
| Transparency in rationale | 18.68 | 1, 575 | 0.00 | 0.03 |  | 7.53 | 1, 403 | 0.01 | 0.02 |  | 55.31 | 1, 698 | 0.00 | 0.07 |  | 6.73 | 1, 590 | 0.01 | 0.01 |
| Predictable framework | 12.23 | 1, 575 | 0.00 | 0.02 |  | 1.87 | 1, 403 | 0.17 | 0.00 |  | 13.26 | 1, 698 | 0.00 | 0.02 |  | 5.41 | 1, 590 | 0.02 | 0.01 |
| Age group | 3.30 | 2, 575 | 0.04 | 0.01 |  | 0.98 | 2, 403 | 0.37 | 0.00 |  | 0.42 | 2, 698 | 0.66 | 0.00 |  | 22.27 | 2, 590 | 0.00 | 0.07 |
| Gender | 0.50 | 3, 575 | 0.68 | 0.00 |  | 2.00 | 1, 403 | 0.16 | 0.00 |  | 0.65 | 3, 698 | 0.58 | 0.00 |  | 2.12 | 2, 590 | 0.12 | 0.01 |
| Generalized interpersonal trust | 33.55 | 1, 575 | 0.00 | 0.06 |  | 63.06 | 1, 403 | 0.00 | 0.14 |  | 12.76 | 1, 698 | 0.00 | 0.02 |  | 24.27 | 1, 590 | 0.00 | 0.04 |
| Voice * Age group | 0.10 | 4, 575 | 0.98 | 0.00 |  | 2.94 | 4, 403 | 0.02 | 0.03 |  | 4.49 | 4, 698 | 0.00 | 0.03 |  | 3.74 | 4, 590 | 0.01 | 0.02 |
| Transparency in rationale * Age group | 1.64 | 2, 575 | 0.20 | 0.01 |  | 0.12 | 2, 403 | 0.89 | 0.00 |  | 3.92 | 2, 698 | 0.02 | 0.01 |  | 12.66 | 2, 590 | 0.00 | 0.04 |
| Predictable framework * Age group | 0.59 | 2, 575 | 0.56 | 0.00 |  | 0.01 | 2, 403 | 0.99 | 0.00 |  | 2.87 | 2, 698 | 0.06 | 0.01 |  | 0.11 | 2, 590 | 0.90 | 0.00 |
| N | 594 |  |  |  |  | 420 |  |  |  |  | 717 |  |  |  |  | 608 |  |  |  |

**4. Pairwise comparisons between experimental conditions by age**

|  | Trust in school management | | | |  | Trust in government | | | |
| --- | --- | --- | --- | --- | --- | --- | --- | --- | --- |
|  | Germany | Italy | Czechia | Serbia |  | Germany | Italy | Czechia | Serbia |
| Citizen voice |  |  |  |  |  |  |  |  |  |
| 11-12 | 0.46 (0.17, 0.76) | 0.42 (0.04, 0.79) | 0.45 (0.19, 0.72) | 0.20 (-0.11, 0.51) |  | 0.46 (0.13, 0.79) | 0.22 (-0.19, 0.63) | 0.33 (0.06, 0.60) | 0.03 (-0.30, 0.36) |
| 14-15 | 0.27 (-0.03, 0.57) | 0.42 (0.08, 0.76) | 0.16 (-0.09, 0.40) | 0.40 (0.07, 0.72) |  | 0.36 (0.04, 0.67) | 0.75 (0.38, 1.11) | 0.64 (0.37, 0.91) | 0.36 (0.04, 0.69) |
| 18-19 | 0.21 (-0.08, 0.50) | 0.08 (-0.25, 0.42) | 0.51 (0.27, 0.74) | 0.37 (0.06, 0.69) |  | 0.48 (0.16, 0.81) | 0.17 (-0.22, 0.55) | 0.63 (0.40, 0.87) | 0.90 (0.58, 1.23) |
| Expert voice |  |  |  |  |  |  |  |  |  |
| 11-12 | 0.39 (0.10, 0.69) | 0.42 (0.02, 0.81) | 0.28 (0.03, 0.54) | 0.36 (0.03, 0.68) |  | 0.46 (0.14, 0.79) | 0.41 (-0.02, 0.85) | 0.10 (-0.17, 0.37) | 0.10 (-0.23, 0.43) |
| 14-15 | 0.48 (0.19, 0.76) | 0.26 (-0.07, 0.59) | 0.15 (-0.10, 0.41) | 0.42 (0.11, 0.74) |  | 0.39 (0.08, 0.71) | 0.11 (-0.26, 0.47) | 0.39 (0.13, 0.65) | 0.15 (-0.19, 0.48) |
| 18-19 | 0.15 (-0.15, 0.44) | 0.08 (-0.27, 0.43) | 0.32 (0.09, 0.54) | 0.50 (0.20, 0.81) |  | 0.43 (0.12, 0.74) | 0.08 (-0.30, 0.46) | 0.82 (0.58, 1.06) | 0.60 (0.28, 0.93) |
| Transparency in rationale |  |  |  |  |  |  |  |  |  |
| 11-12 | 0.36 (0.12, 0.61) | 0.33 (0.01, 0.64) | 0.09 (-0.12, 0.30) | 0.09 (-0.17, 0.35) |  | 0.37 (0.11, 0.64) | 0.31 (-0.03, 0.65) | 0.28 (0.06, 0.50) | -0.14 (-0.40, 0.13) |
| 14-15 | 0.31 (0.07, 0.55) | 0.47 (0.19, 0.75) | 0.29 (0.09, 0.50) | 0.24 (-0.03, 0.50) |  | 0.15 (-0.12, 0.41) | 0.20 (-0.10, 0.49) | 0.42 (0.20, 0.63) | 0.00 (-0.27, 0.27) |
| 18-19 | 0.29 (0.05, 0.53) | -0.07 (-0.36, 0.22) | 0.49 (0.30, 0.67) | 0.55 (0.29, 0.81) |  | 0.48 (0.22, 0.74) | 0.26 (-0.05, 0.56) | 0.69 (0.49, 0.88) | 0.75 (0.49, 1.01) |
| Predictable framework |  |  |  |  |  |  |  |  |  |
| 11-12 | 0.32 (0.07, 0.56) | 0.29 (-0.03, 0.61) | 0.16 (-0.05, 0.37) | 0.14 (-0.12, 0.40) |  | 0.28 (0.02, 0.55) | 0.14 (-0.20, 0.48) | 0.01 (-0.21, 0.23) | 0.15 (-0.12, 0.41) |
| 14-15 | 0.06 (-0.18, 0.31) | 0.24 (-0.04, 0.52) | 0.29 (0.09, 0.50) | 0.21 (-0.05, 0.48) |  | 0.36 (0.10, 0.62) | 0.12 (-0.17, 0.42) | 0.36 (0.15, 0.58) | 0.17 (-0.10, 0.44) |
| 18-19 | 0.01 (-0.23, 0.25) | 0.16 (-0.13, 0.45) | 0.10 (-0.08, 0.29) | 0.52 (0.27, 0.78) |  | 0.16 (-0.09, 0.42) | 0.11 (-0.19, 0.42) | 0.30 (0.10, 0.49) | 0.23 (-0.03, 0.49) |

**5. Analytical code – measurement invariance testing**

***Exporting key variables from the .sav format in SPSS***

WRITE OUTFILE='trust_data.dat'

ENCODING='UTF8'

TABLE

/country age_cat vman_t1 vman_t2 vman_t3 vman_t4 vgov_t1 vgov_t2 vgov_t3 vgov_t4.

EXECUTE.

***Mplus code:***

DATA: FILE IS trust_data.dat; FORMAT IS 10F1.0;

VARIABLE: NAMES ARE ctry age s1 s2 s3 s4 g1 g2 g3 g4;

USEOBSERVATIONS IS (ctry EQ 1); ! 1=GER 2=ITA 3=CZE 4=SRB

USEVARIABLES IS s1-g4;

!USEVARIABLES IS s1 s2 s4-g4; !3-item version of school trust

MISSING ARE BLANK;

GROUPING IS age (1=early 2=middle 3=late);

ANALYSIS: ESTIMATOR=WLSMV;

MODEL = CONFIGURAL METRIC SCALAR; !not use when inspecting modindices

MODEL:

s BY s1-s4;

!s BY s1 s2 s4; !3-item version of school trust

g BY g1-g4;

s1 WITH g1;

s2 WITH g2;

s3 WITH g3; ! not use for the 3-item version of school trust

s4 WITH g4;

! metric model !use when inspecting modindices for the metric model

!MODEL early:

![s1-s4*]; [g1-g4*]; [s@0];[g@0];

!MODEL middle:

![s1-s4*]; [g1-g4*]; [s@0];[g@0];

! partial invariance in SRB

!MODEL late: [g1*];[g2*];[s4*];

!OUTPUT: modindices (4); !use when inspecting modindices

**5. Analytical code – the main analysis**

***R code:***

library(haven)

library(dplyr)

library(car)

library(rstatix)

library(emmeans)

library(ggplot2)

library(patchwork)

# reading the data

data<- read_spss("trust_data.sav")

data$age_cat<-factor(data$age_cat)

data$gndr<-factor(data$gndr)

data$vman_voice<-factor(data$vman_voice)

data$vman_tran<-factor(data$vman_tran)

data$vman_pred<-factor(data$vman_pred)

data$vgov_voice<-factor(data$vgov_voice)

data$vgov_tran<-factor(data$vgov_tran)

data$vgov_pred<-factor(data$vgov_pred)

data_ger<-data %>%

filter(country==1)

data_ita<-data %>%

filter(country==2)

data_cze<-data %>%

filter(country==3)

data_srb<-data %>%

filter(country==4)

data_use<-data %>%

filter(country==3)

# anova intro

options(contrasts = c("contr.helmert", "contr.poly"))

# anova - school - no interactions

model_man_noint_ger <- lm(vman_trust ~ vman_voice + vman_tran + vman_pred + age_cat + gen_trust + gndr, data=data_ger)

anova_man_noint_ger <- Anova(model_man_noint_ger, type="III")

eta_man_noint_ger <- partial_eta_squared(anova_man_noint_ger)

emm_voice_man_noint_ger <- emmeans(model_man_noint_ger, ~ vman_voice)

pairwise_voice_man_noint_ger <- pairs(emm_voice_man_noint_ger, adjust = "bonferroni")

model_man_noint_ita <- lm(vman_trust ~ vman_voice + vman_tran + vman_pred + age_cat + gen_trust + gndr, data=data_ita)

anova_man_noint_ita <- Anova(model_man_noint_ita, type="III")

eta_man_noint_ita <- partial_eta_squared(anova_man_noint_ita)

emm_voice_man_noint_ita <- emmeans(model_man_noint_ita, ~ vman_voice)

pairwise_voice_man_noint_ita <- pairs(emm_voice_man_noint_ita, adjust = "bonferroni")

model_man_noint_cze <- lm(vman_trust ~ vman_voice + vman_tran + vman_pred + age_cat + gen_trust + gndr, data=data_cze)

anova_man_noint_cze <- Anova(model_man_noint_cze, type="III")

eta_man_noint_cze <- partial_eta_squared(anova_man_noint_cze)

emm_voice_man_noint_cze <- emmeans(model_man_noint_cze, ~ vman_voice)

pairwise_voice_man_noint_cze <- pairs(emm_voice_man_noint_cze, adjust = "bonferroni")

model_man_noint_srb <- lm(vman_trust ~ vman_voice + vman_tran + vman_pred + age_cat + gen_trust + gndr, data=data_srb)

anova_man_noint_srb <- Anova(model_man_noint_srb, type="III")

eta_man_noint_srb <- partial_eta_squared(anova_man_noint_srb)

emm_voice_man_noint_srb <- emmeans(model_man_noint_srb, ~ vman_voice)

pairwise_voice_man_noint_srb <- pairs(emm_voice_man_noint_srb, adjust = "bonferroni")

print(anova_man_noint_ger)

print(anova_man_noint_ita)

print(anova_man_noint_cze)

print(anova_man_noint_srb)

print(eta_man_noint_ger)

print(eta_man_noint_ita)

print(eta_man_noint_cze)

print(eta_man_noint_srb)

nobs(model_man_noint_ger)

nobs(model_man_noint_ita)

nobs(model_man_noint_cze)

nobs(model_man_noint_srb)

print(pairwise_voice_man_noint_ger)

print(pairwise_voice_man_noint_ita)

print(pairwise_voice_man_noint_cze)

print(pairwise_voice_man_noint_srb)

# anova - school - interactions

model_man_ger <- lm(vman_trust ~ vman_voice + vman_tran + vman_pred + age_cat + gen_trust + gndr

+ vman_voice * age_cat + vman_tran * age_cat + vman_pred * age_cat, data=data_ger)

anova_man_ger <- Anova(model_man_ger, type="III")

eta_man_ger <- partial_eta_squared(anova_man_ger)

model_man_ita <- lm(vman_trust ~ vman_voice + vman_tran + vman_pred + age_cat + gen_trust + gndr

+ vman_voice * age_cat + vman_tran * age_cat + vman_pred * age_cat, data=data_ita)

anova_man_ita <- Anova(model_man_ita, type="III")

eta_man_ita <- partial_eta_squared(anova_man_ita)

model_man_cze <- lm(vman_trust ~ vman_voice + vman_tran + vman_pred + age_cat + gen_trust + gndr

+ vman_voice * age_cat + vman_tran * age_cat + vman_pred * age_cat, data=data_cze)

anova_man_cze <- Anova(model_man_cze, type="III")

eta_man_cze <- partial_eta_squared(anova_man_cze)

model_man_srb <- lm(vman_trust ~ vman_voice + vman_tran + vman_pred + age_cat + gen_trust + gndr

+ vman_voice * age_cat + vman_tran * age_cat + vman_pred * age_cat, data=data_srb)

anova_man_srb <- Anova(model_man_srb, type="III")

eta_man_srb <- partial_eta_squared(anova_man_srb)

print(anova_man_ger)

print(anova_man_ita)

print(anova_man_cze)

print(anova_man_srb)

print(eta_man_ger)

print(eta_man_ita)

print(eta_man_cze)

print(eta_man_srb)

nobs(model_man_ger)

nobs(model_man_ita)

nobs(model_man_cze)

nobs(model_man_srb)

# anova - government - no interactions

model_gov_noint_ger <- lm(vgov_trust ~ vgov_voice + vgov_tran + vgov_pred + age_cat + gndr + gen_trust, data=data_ger)

anova_gov_noint_ger <- Anova(model_gov_noint_ger, type="III")

eta_gov_noint_ger <- partial_eta_squared(anova_gov_noint_ger)

emm_voice_gov_noint_ger <- emmeans(model_gov_noint_ger, ~ vgov_voice)

pairwise_voice_gov_noint_ger <- pairs(emm_voice_gov_noint_ger, adjust = "bonferroni")

model_gov_noint_ita <- lm(vgov_trust ~ vgov_voice + vgov_tran + vgov_pred + age_cat + gndr + gen_trust, data=data_ita)

anova_gov_noint_ita <- Anova(model_gov_noint_ita, type="III")

eta_gov_noint_ita <- partial_eta_squared(anova_gov_noint_ita)

emm_voice_gov_noint_ita <- emmeans(model_gov_noint_ita, ~ vgov_voice)

pairwise_voice_gov_noint_ita <- pairs(emm_voice_gov_noint_ita, adjust = "bonferroni")

model_gov_noint_cze <- lm(vgov_trust ~ vgov_voice + vgov_tran + vgov_pred + age_cat + gndr + gen_trust, data=data_cze)

anova_gov_noint_cze <- Anova(model_gov_noint_cze, type="III")

eta_gov_noint_cze <- partial_eta_squared(anova_gov_noint_cze)

emm_voice_gov_noint_cze <- emmeans(model_gov_noint_cze, ~ vgov_voice)

pairwise_voice_gov_noint_cze <- pairs(emm_voice_gov_noint_cze, adjust = "bonferroni")

model_gov_noint_srb <- lm(vgov_trust ~ vgov_voice + vgov_tran + vgov_pred + age_cat + gndr + gen_trust, data=data_srb)

anova_gov_noint_srb <- Anova(model_gov_noint_srb, type="III")

eta_gov_noint_srb <- partial_eta_squared(anova_gov_noint_srb)

emm_voice_gov_noint_srb <- emmeans(model_gov_noint_srb, ~ vgov_voice)

pairwise_voice_gov_noint_srb <- pairs(emm_voice_gov_noint_srb, adjust = "bonferroni")

print(anova_gov_noint_ger)

print(anova_gov_noint_ita)

print(anova_gov_noint_cze)

print(anova_gov_noint_srb)

print(eta_gov_noint_ger)

print(eta_gov_noint_ita)

print(eta_gov_noint_cze)

print(eta_gov_noint_srb)

nobs(model_gov_noint_ger)

nobs(model_gov_noint_ita)

nobs(model_gov_noint_cze)

nobs(model_gov_noint_srb)

print(pairwise_voice_gov_noint_ger)

print(pairwise_voice_gov_noint_ita)

print(pairwise_voice_gov_noint_cze)

print(pairwise_voice_gov_noint_srb)

# anova - government - interactions

model_gov_ger <- lm(vgov_trust ~ vgov_voice + vgov_tran + vgov_pred + age_cat + gndr + gen_trust

+ vgov_voice * age_cat + vgov_tran * age_cat + vgov_pred * age_cat, data=data_ger)

anova_gov_ger <- Anova(model_gov_ger, type="III")

eta_gov_ger <- partial_eta_squared(anova_gov_ger)

model_gov_ita <- lm(vgov_trust ~ vgov_voice + vgov_tran + vgov_pred + age_cat + gndr + gen_trust

+ vgov_voice * age_cat + vgov_tran * age_cat + vgov_pred * age_cat, data=data_ita)

anova_gov_ita <- Anova(model_gov_ita, type="III")

eta_gov_ita <- partial_eta_squared(anova_gov_ita)

model_gov_cze <- lm(vgov_trust ~ vgov_voice + vgov_tran + vgov_pred + age_cat + gndr + gen_trust

+ vgov_voice * age_cat + vgov_tran * age_cat + vgov_pred * age_cat, data=data_cze)

anova_gov_cze <- Anova(model_gov_cze, type="III")

eta_gov_cze <- partial_eta_squared(anova_gov_cze)

model_gov_srb <- lm(vgov_trust ~ vgov_voice + vgov_tran + vgov_pred + age_cat + gndr + gen_trust

+ vgov_voice * age_cat + vgov_tran * age_cat + vgov_pred * age_cat, data=data_srb)

anova_gov_srb <- Anova(model_gov_srb, type="III")

eta_gov_srb <- partial_eta_squared(anova_gov_srb)

print(anova_gov_ger)

print(anova_gov_ita)

print(anova_gov_cze)

print(anova_gov_srb)

print(eta_gov_ger)

print(eta_gov_ita)

print(eta_gov_cze)

print(eta_gov_srb)

nobs(model_gov_ger)

nobs(model_gov_ita)

nobs(model_gov_cze)

nobs(model_gov_srb)

# simple effects - age_cat*voice

emm_ita <- emmeans(model_gov_ita, ~ vgov_voice * age_cat)

seff_ita_citizens <- contrast(emm_ita,

method = list("0 vs 2" = c(-1, 1, 0)),

by = "age_cat")

joint_tests(seff_ita_citizens, by = NULL)

seff_ita_experts <- contrast(emm_ita,

method = list("0 vs 2" = c(-1, 0, 1)),

by = "age_cat")

joint_tests(seff_ita_experts, by = NULL)

emm_cze <- emmeans(model_gov_cze, ~ vgov_voice * age_cat)

seff_cze_citizens <- contrast(emm_cze,

method = list("0 vs 2" = c(-1, 1, 0)),

by = "age_cat")

joint_tests(seff_cze_citizens, by = NULL)

seff_cze_experts <- contrast(emm_cze,

method = list("0 vs 2" = c(-1, 0, 1)),

by = "age_cat")

joint_tests(seff_cze_experts, by = NULL)

emm_srb <- emmeans(model_gov_srb, ~ vgov_voice * age_cat)

seff_srb_citizens <- contrast(emm_srb,

method = list("0 vs 2" = c(-1, 1, 0)),

by = "age_cat")

joint_tests(seff_srb_citizens, by = NULL)

seff_srb_experts <- contrast(emm_srb,

method = list("0 vs 2" = c(-1, 0, 1)),

by = "age_cat")

joint_tests(seff_srb_experts, by = NULL)

# contrasts - school

get_contrasts_man <- function(model, sample_name){

contrast_voice <- contrast(emmeans(model, ~ vman_voice | age_cat), method = "revpairwise", adjust = "none")

contrast_tran <- contrast(emmeans(model, ~ vman_tran | age_cat), method = "revpairwise", adjust = "none")

contrast_pred <- contrast(emmeans(model, ~ vman_pred | age_cat), method = "revpairwise", adjust = "none")

df_voice <- as.data.frame(confint(contrast_voice)) %>%

mutate(predictor = "Voice")

df_tran <- as.data.frame(confint(contrast_tran)) %>%

mutate(predictor = "Transparency")

df_pred <- as.data.frame(confint(contrast_pred)) %>%

mutate(predictor = "Predictability")

bind_rows(df_voice, df_tran, df_pred) %>%

mutate(sample = sample_name) %>%

select(sample, predictor, age_cat, contrast, estimate, SE, df, lower.CL, upper.CL)

}

effects_man <- bind_rows(

get_contrasts_man(model_man_ger, "GER"),

get_contrasts_man(model_man_ita, "ITA"),

get_contrasts_man(model_man_cze, "CZE"),

get_contrasts_man(model_man_srb, "SRB")

)

effects_man_clean <- effects_man %>%

filter(contrast != "vman_voice2 - vman_voice1")

effects_man_clean <- effects_man_clean %>%

mutate(

predictor = case_when(

predictor == "Voice" & contrast == "vman_voice1 - vman_voice0" ~ "Voice citizens",

predictor == "Voice" & contrast == "vman_voice2 - vman_voice0" ~ "Voice experts",

TRUE ~ predictor

),

sample = factor(

sample,

levels = c("GER", "ITA", "CZE", "SRB")

),

predictor = factor(

predictor,

levels = c("Voice citizens", "Voice experts", "Transparency", "Predictability")

),

age_cat = factor(

age_cat,

levels = c("1", "2", "3")

)

) %>%

arrange(sample, predictor, age_cat)

print(effects_man_clean)

# contrasts - government

get_contrasts_gov <- function(model, sample_name){

contrast_voice <- contrast(emmeans(model, ~ vgov_voice | age_cat), method = "revpairwise", adjust = "none")

contrast_tran <- contrast(emmeans(model, ~ vgov_tran | age_cat), method = "revpairwise", adjust = "none")

contrast_pred <- contrast(emmeans(model, ~ vgov_pred | age_cat), method = "revpairwise", adjust = "none")

df_voice <- as.data.frame(confint(contrast_voice)) %>%

mutate(predictor = "Voice")

df_tran <- as.data.frame(confint(contrast_tran)) %>%

mutate(predictor = "Transparency")

df_pred <- as.data.frame(confint(contrast_pred)) %>%

mutate(predictor = "Predictability")

bind_rows(df_voice, df_tran, df_pred) %>%

mutate(sample = sample_name) %>%

select(sample, predictor, age_cat, contrast, estimate, SE, df, lower.CL, upper.CL)

}

effects_gov <- bind_rows(

get_contrasts_gov(model_gov_ger, "GER"),

get_contrasts_gov(model_gov_ita, "ITA"),

get_contrasts_gov(model_gov_cze, "CZE"),

get_contrasts_gov(model_gov_srb, "SRB")

)

effects_gov_clean <- effects_gov %>%

filter(contrast != "vgov_voice2 - vgov_voice1")

effects_gov_clean <- effects_gov_clean %>%

mutate(

predictor = case_when(

predictor == "Voice" & contrast == "vgov_voice1 - vgov_voice0" ~ "Voice citizens",

predictor == "Voice" & contrast == "vgov_voice2 - vgov_voice0" ~ "Voice experts",

TRUE ~ predictor

),

sample = factor(

sample,

levels = c("GER", "ITA", "CZE", "SRB")

),

predictor = factor(

predictor,

levels = c("Voice citizens", "Voice experts", "Transparency", "Predictability")

),

age_cat = factor(

age_cat,

levels = c("1", "2", "3")

)

) %>%

arrange(sample, predictor, age_cat)

print(effects_gov_clean)

# plots

school_plot <- ggplot(

effects_man_clean,

aes(x = sample, y = estimate,

ymin = lower.CL, ymax = upper.CL,

color = age_cat, group = age_cat)

) +

geom_pointrange(position = position_dodge(width = 0.6)) +

geom_hline(yintercept = 0, linetype = "dashed") +

facet_wrap(~ predictor, nrow = 1,

labeller = labeller(

predictor = c(

"Voice citizens" = "Citizen voice",

"Voice experts" = "Expert voice",

"Transparency" = "Transparency in rationale",

"Predictability" = "Predictable framework"

)

)) +

scale_color_grey(start = 0.2, end = 0.7,

labels = c("11-12", "14-15", "18-19")) +

labs(

x = NULL,

y = "Trust in school management",

color = "Age group"

) +

theme_bw(base_size = 14) +

theme(

legend.position = "bottom",

strip.text = element_text(face = "bold")

)

gov_plot <- ggplot(

effects_gov_clean,

aes(x = sample, y = estimate,

ymin = lower.CL, ymax = upper.CL,

color = age_cat, group = age_cat)

) +

geom_pointrange(position = position_dodge(width = 0.6)) +

geom_hline(yintercept = 0, linetype = "dashed") +

facet_wrap(~ predictor, nrow = 1,

labeller = labeller(

predictor = c(

"Voice citizens" = "Citizen voice",

"Voice experts" = "Expert voice",

"Transparency" = "Transparency in rationale",

"Predictability" = "Predictable framework"

)

)) +

scale_color_grey(start = 0.2, end = 0.7,

labels = c("11-12", "14-15", "18-19")) +

labs(

x = NULL,

y = "Trust in government",

color = "Age group"

) +

theme_bw(base_size = 14) +

theme(

legend.position = "bottom",

strip.text = element_text(face = "bold")

)

combined_plot <- school_plot / gov_plot +

plot_layout(guides = "collect") & theme(legend.position = "bottom")

combined_plot
